# Pirfenidone Is a Vasodilator: Involvement of K_V_7 Channels in the Effect on Endothelium-Dependent Vasodilatation in Type-2 Diabetic Mice

**DOI:** 10.3389/fphar.2020.619152

**Published:** 2021-01-12

**Authors:** Lilliana Beck, Estéfano Pinilla, Daniel Dias Rufino Arcanjo, Raquel Hernanz, Judit Prat-Duran, Asbjørn Graver Petersen, Ralf Köhler, Majid Sheykhzade, Simon Comerma-Steffensen, Ulf Simonsen

**Affiliations:** ^1^ Department of Biomedicine, Pulmonary and Cardiovascular Pharmacology, Aarhus University, Aarhus, Denmark; ^2^ Department of Physiology, Faculty of Pharmacy, Universidad Complutense, Madrid, Spain; ^3^ Department of Biophysics and Physiology, Laboratory of Functional and Molecular Studies in Physiopharmacology, Federal University of Piauí, Teresina, Brazil; ^4^ Departamento de Ciencias Básicas de la Salud, Universidad Rey Juan Carlos, Alcorcón, Spain; ^5^ Aragón Agency for Research and Development (ARAID), Zaragoza, Spain; ^6^ Department of Drug Design and Pharmacology, Faculty of Health and Medical Sciences, University of Copenhagen, Copenhagen, Denmark; ^7^ Department of Biomedical Sciences/Animal Physiology, Faculty of Veterinary, Central University of Venezuela, Maracay, Venezuela

**Keywords:** endothelium, coronary arteries, pirfenidone, pulmonary arteries, mouse aorta, type 2 diabetes, large-conductance calcium-activated K channels, voltage-gated KV7 channels

## Abstract

Endothelial cell dysfunction and fibrosis are associated with worsening of the prognosis in patients with cardiovascular disease. Pirfenidone has a direct antifibrotic effect, but vasodilatation may also contribute to the effects of pirfenidone. Therefore, in a first study we investigated the mechanisms involved in the relaxant effect of pirfenidone in rat intrapulmonary arteries and coronary arteries from normal mice. Then in a second study, we investigated whether pirfenidone restores endothelial function in the aorta and mesenteric arteries from diabetic animals. From 16–18-week old normal male C57BL/6 mice and normoglycemic (db/db+), and type 2 diabetic (db/db) male and female mice, arteries were mounted in microvascular isometric myographs for functional studies, and immunoblotting was performed. In rat pulmonary arteries and mouse coronary arteries, pirfenidone induced relaxations, which were inhibited in preparations without endothelium. In mouse coronary arteries, pirfenidone relaxation was inhibited in the presence of a nitric oxide (NO) synthase inhibitor, N^G^-nitro-l-arginine (L-NOARG), a blocker of large-conductance calcium-activated potassium channels (BK_Ca_), iberiotoxin, and a blocker of K_V_7 channels, XE991. Patch clamp studies in vascular smooth muscle revealed pirfenidone increased iberiotoxin-sensitive current. In the aorta and mesenteric small arteries from diabetic db/db mice relaxations induced by the endothelium-dependent vasodilator, acetylcholine, were markedly reduced compared to db/db + mice. Pirfenidone enhanced the relaxations induced by acetylcholine in the aorta from diabetic male and female db/db mice. An opener of K_V_7 channels, flupirtine, had the same effect as pirfenidone. XE991 reduced the effect of pirfenidone and flupirtine and further reduced acetylcholine relaxations in the aorta. In the presence of iberiotoxin, pirfenidone still increased acetylcholine relaxation in aorta from db/db mice. Immunoblotting for K_V_7.4, K_V_7.5, and BK_Ca_ channel subunits were unaltered in aorta from db/db mice. Pirfenidone failed to improve acetylcholine relaxation in mesenteric arteries, and neither changed acetylcholine-induced transient decreases in blood pressure in db/db+ and db/db mice. In conclusion, pirfenidone vasodilates pulmonary and coronary arteries. In coronary arteries from normal mice, pirfenidone induces NO-dependent vasodilatation involving BK_Ca_ and K_V_7 channels. Pirfenidone improves endothelium-dependent vasodilatation in aorta from diabetic animals by a mechanism involving voltage-gated K_V_7 channels, a mechanism that may contribute to the antifibrotic effect of pirfenidone.

## Introduction

Pirfenidone is used for the treatment of pulmonary fibrosis and modulates fibrogenesis by inhibition of transforming growth factor (TGFβ) activity (Iyer et al., 1999; Hilberg et al., 2012). Moreover, pirfenidone has also been shown to have cardioprotective effects by reducing ventricular fibrosis in preclinical studies (Aimo et al., 2020). Pirfenidone is ineffective as superoxide radical scavenger (Gaggini et al., 2011), but scavenges hydroxyl radicals (Misra and Rabideau, 2000), although the plasma concentrations of 8–9 mg/L obtained in patients with idiopathic pulmonary fibrosis are in the low end of the hydroxyl scavenging effect of pirfenidone (Hilberg et al., 2012). However, low micromolar concentrations of pirfenidone potentiated L-type calcium current in rat cardiac myocytes (Ramos-Mondragón et al., 2012). Therefore, other mechanisms may contribute to the antifibrotic mechanism of pirfenidone.

Pirfenidone has a direct antifibrotic effect, but vasodilatation may also contribute to the antifibrotic and anti-inflammatory effects of pirfenidone. In preliminary studies in pulmonary arteries we observed pirfenidone induces relaxation, and in recent studies in acute kidney injury induced by ischemia-reperfusion, pirfenidone restored renal blood flow and increased the formation of nitric oxide (NO) products (Lima-Posada et al., 2019). Based on these observations, and due to the emerging role of pirfenidone in cardioprotection, we in a first study investigated the mechanisms involved in the relaxant effect of pirfenidone in coronary arteries, and found that potassium channels were involved in the vasodilatation.

Genetic disposition, hyperglycemia, production of advanced glycation end products, hyperlipidemia, insulin resistance, and hypertension are important for the development of cardiovascular disease in type 2 diabetes (Rydén et al., 2013). The bioavailability and effects of nitric oxide (NO) and endothelium-derived hyperpolarization (EDH) have been reported to be impaired in arteries from type 2 diabetic rats and mice (Bagi et al., 2005; Pannirselvam et al., 2005; Gao et al., 2007; Park et al., 2008; Zhang et al., 2008; Brøndum et al., 2010, Bagi et al., 2015; Lee et al., 2017). Decreased bioavailability of NO due to upregulation of tumor necrosis factor (TNFalpha) and increased formation of free radical species is considered an important mechanism for arterial endothelial dysfunction in diabetic animals (Beckman et al., 2001; Pannirselvam et al., 2002; Gao et al., 2007; Zhang et al., 2008). Treatment with antioxidants seems to restore not only NO bioavailability (Zhang et al., 2008), but also the EDH pathway in small arteries from diabetic animals (Chen et al., 2015). Despite, the effect of antioxidants in clinical trials remains limited (Sesso et al., 2008), there are already several drugs with other mechanisms of action that are also claimed to be antioxidants including carvedilol (Abreu et al., 2000; Dandona et al., 2007) and the antifibrotic drug, pirfenidone (Misra and Rabideau, 2000).

In addition to antioxidants, modulation of endothelial small-conductance (SK_Ca_ or K_Ca_2.3) and intermediate-conductance calcium-activated potassium (IK_Ca_ or K_Ca_3.1) channels partially restore endothelial function in arteries from diabetic animals (Brøndum et al., 2010). Modulation of smooth muscle large-conductance calcium-activated potassium channels (BK_Ca_) and K_V_ channels was also suggested for improving endothelium-dependent vasodilatation (Félétou, 2009). The lipid-lowering antioxidant probucol restores contribution of BK_Ca_ channels to β_2_-adrenoceptor vasodilation in retinal arteries from diabetic rats (Mori et al., 2017), while activation of peroxisome proliferator-activated receptor-b/d (PPARβ/δ) prevented hyperglycaemia-induced impairment of K_V_7 channels and lowered oxidative stress in coronary arteries from rats with streptozocin-induced diabetes (Morales-Cano et al., 2016). Therefore, we hypothesized in a second study that the antifibrotic drug, pirfenidone improves endothelium-dependent vasodilation in large and small systemic arteries from diabetic animals. Diabetic db/db mice develop obesity and hyperglycaemia similar to that seen in human type 2 diabetes due to a spontaneous mutation in the leptin receptor gene. Therefore, we examined in the aorta from diabetic db/db mice whether the potassium channels involved in the direct coronary vasodilator effect also contribute to the increases in acetylcholine vasorelaxation induced by pirfenidone.

## Methods and Material

### Animals and Preparation of Samples

All animal experiments in this study were conducted under the supervision of a veterinarian and following the Danish legislation of animal use for scientific procedures as described in the “Animal Testing Act” (Consolidation Act No. 726 of September 9, 1993 as amended by Act No. 1081 of December 20, 1995). They were approved by the Danish Animal Experiments Inspectorate (permission 2014-15-2934-01059) and reported by following the ARRIVE guidelines (McGrath and Lilley, 2015). The Danish “Animal Testing Act” fully and extensively covered the requirements included in the Guide for the Care and Use of Laboratory Animals as adopted and promulgated by the U.S. National Health Institute. The animals were housed in the animal facility in 365 × 207 × 140 mm cages with standard wood bedding and space for one rat or three to four mice. Adult male Wistar rats (10–12-weeks old) of the Hannover strain and weighing 300–350 g were obtained from Taconic, Ry, Denmark. The rats were housed in cages (Techniplast, Buguggiate, Italy, 800 cm^2^) filled with aspen woodchips (Tapvei, Datesand, Manchester, United Kingdom) and nesting material (Soft Paper Wool/LBS, United Kingdom) as bedding. The animals had free access to food (Brogaarden, Middelfart, DK) and drinking water. The animals had a 12-h light/dark cycle, and to avoid influence of torpor (Swoap and Gutilla, 2009; Ayala et al., 2010), the mice were allocated to experimentation early in the light period.

The animals were selected randomly, and wherever possible, observations were made without knowledge of the treatments administered. Body weight and blood glucose levels of the animals were measured before sacrificing the animals. Normal male C57BL/6 mice, and male and female mice of the breed db/db (C57BLKS/J-lepr^db^/lepr^db^) and age-matched db/db+ mice (C57BLKS/J-lepr^db^) (Taconic, Ry, Denmark) were euthanized by cervical dislocation. The heart, aorta, and intestine were immediately removed and placed in a 4 °C physiological saline solution (PSS, pH = 7.4) of the following composition (mM): 119 NaCl, 4.7 KCl, 1.18 KH_2_PO_4_, 1.17 MgSO_4_, 1.5 CaCl_2_, 24.9 NaHCO_3_, 0.026 EDTA, and 5.5 glucose. To avoid acute hyperglycaemic effects on endothelial function (Bangshaab et al., 2019), which may confound the relation of endothelial to structural function, experiments on arteries from both control db/db+ and diabetic db/db male and female mice were conducted with the same glucose concentration.

### Investigation of the Direct Vasodilator Effect of Pirfenidone

#### Functional Studies in Rat Pulmonary Arteries

Segments of intrapulmonary pulmonary arteries approximately 2 mm long were carefully dissected from the lung of male Wistar Hannover rats as previously described (Kroigaard et al., 2013). Adherent tissue was removed. Subsequently, the pulmonary arteries were mounted on two 40 μm steel wires in microvascular myographs (Danish Myotechnology, Aarhus, Denmark) for isometric tension recordings. The pulmonary arteries were placed in organ baths with PSS, and the baths were heated to 37 °C and equilibrated with 5% CO_2_ in bioair = 21% O_2_, 74% N_2_ to maintain the desired pH of 7.4. The segments were then equilibrated for 10 min. For optimal measurements, pulmonary arteries were stretched to 3.9 kPa, corresponding to a transmural pressure of 30 mmHg (Kroigaard et al., 2013). After 10 min equilibration, potassium-rich PSS (60 mM KPSS) was added twice to test the viability of the segments. KPSS was equivalent to PSS but NaCl exchanged with KCl on an equimolar basis. The pulmonary artery endothelial function was tested by contracting the segments using the thromboxane analogue 9,11-dideoxy-9a, 11a-epoxymethanoprostaglandin F_2α_ (U46619, 0.03 μM) and then adding ACh (10 μM). Only segments with more than 50% relaxation in response to ACh were included in the study. In a series of experiments, the endothelium was removed by rubbing a small hair against the inner surface of the segment. The absence of endothelium was confirmed by relaxation in response to ACh of less than 10%.

The segments were contracted with U46619, and concentration-response curves for pirfenidone, the hydroxyl scavenger mannitol, and the superoxide scavenger, tempol were constructed. To investigate the role of the endothelium, concentration response curves for pirfenidone were constructed in preparations with and without endothelium, or contracted with 30 mM KPSS and increasing concentrations of pirfenidone was added.

#### Functional Studies in Mouse Small Coronary Arteries

From C57BL/6 wild type male mice of 16–18 weeks, coronary small arteries with a length of approximately 2 mm were mounted on two 25-μm wires in microvascular myographs (Danish Myotechnology, Aarhus, Denmark) for isometric tension recordings. The arterial segments were stretched to their optimal diameter, which corresponded to an internal circumference of 90% of that achieved when the vessels were exposed to a passive tension yielding a transmural pressure of 100 mmHg (Simonsen et al., 1999). The baths were heated to 37 °C, and the buffer was equilibrated with 5% CO_2_ in the air to maintain the desired pH of 7.4. The viability of the segments was confirmed by their ability to contract to first potassium-rich PSS (KPSS, 60 mM) and subsequently to the thromboxane analog U46619 (10^−7^ M). To examine endothelial function, acetylcholine (10^−6^ M) was added in U46619-contracted preparations. Only preparations responding with relaxations larger than 40% to acetylcholine were accepted for further experimentation, as previous studies have shown that this corresponds to intact endothelial cell layer in intact coronary arteries (Simonsen et al., 1992; Christensen et al., 2007).

To investigate whether NO and prostanoids are involved in pirfenidone relaxation preparations were incubated with an inhibitor of NO synthase, N^G^-nitro-l-arginine (L-NOARG, 1 mM), or L-NOARG and an inhibitor of cyclooxygenase, indomethacin (3 × 10^−6^ M), and concentration-response curves were performed for pirfenidone in U46619-contracted preparations. To investigate whether potassium channels are involved in pirfenidone relaxation, the coronary arteries were contracted with U46619 (10^−7^ M) or KPSS (30 mM) and when the contraction was stable, increasing concentrations of pirfenidone (10^−6^-3 × 10^−4^ M) or vehicle was added. To investigate the subtypes of potassium channels involved in pirfenidone relaxation, the coronary arteries were incubated for 30 min with: 1) a general blocker of calcium-activated potassium channels, tetraethylammonium (1 mM), 2) a blocker of BK_Ca_ channels iberiotoxin (10^−7^ M), 3) a blocker of IK_Ca_ channels, TRAM-34, or a combination of blockers of SK_Ca_ and IK_Ca_, UCL1684 (10^−6^ M) and TRAM-34 (10^−6^ M), 4) a blocker of voltage-gated potassium channels, K_V_7 channels, XE991 (10^−5^ M), or 5) vehicle, and then the coronary arteries were contracted with U46619 and concentration-response curves performed for pirfenidone.

#### Investigation of Hydroxyl Scavenging Properties of Pirfenidone

It has been proposed that pirfenidone exerts some of its beneficial effects through scavenging of ^•^OH (Misra and Rabideau, 2000). However, so far the ^•^OH scavenging effect of pirfenidone has only been analyzed in a concentration range that is greater than the therapeutic relevant concentration. By using the deoxyribose degradation assay, it is possible to analyze the scavenging effect and determine the second-order rate constant of a drug interacting with ^•^OH (Halliwell et al., 1987). The basis of this assay is the degradation of the sugar deoxyribose upon exposure to ^•^OH generated by a Fenton type reaction. When the reaction mixture is heated under acidic conditions, malonaldehyde (MDA) is formed. The MDA products react with thiobarbituric acid (TBA) and a pink chromogen is formed. The change in color can be measured spectrophotometrically. To generate ^•^OH radicals, a Fe^2+^-EDTA chelate is incubated with deoxyribose in a phosphate buffer at pH 7.4:

$${\text{Fe}}^{2+}-\text{EDTA}+\;2{\text{O}}_{2}↔{\text{Fe}}^{3+}-\text{EDTA}+2{\text{O}}_{2}^{•-}$$

$$2{\text{O}}_{2}^{•-}+2{\text{H}}^{+}\;\to {\text{H}}_{2}{\text{O}}_{2}+{\text{O}}_{2}$$

Fe2+−EDTA+H2O2→OH−+O•H+Fe3+−EDTA

The first step is oxidation of the ferrous-ion into a ferric-ion creating a superoxide radical. This superoxide radical then reacts with hydrogen, forming hydrogen peroxide and molecular oxygen. The second step produces an ^•^OH by the reaction between the ferrous-ion and hydrogen peroxide. Any ^•^OH that escape scavenging by EDTA may react with the deoxyribose:

O•H+deoxyribose→fragments→heat with TBA plus acidMDA

2TBA+MDA→pink chromogen

By adding a drug capable of reacting with the ^•^OH, it will compete with the deoxyribose for the available radicals, decreasing the degradation of deoxyribose. The competition will be dependent of the drug concentration and rate constant of the reaction with ^•^OH. Under the assumption that the rate determining step, of the fragment formation leading to MDA production, is the initial attack by ^•^OH on deoxyribose. The calculation of the rate constant between a scavenger and ^•^OH can be carried out from the analysis of a simple competition between the scavenger and deoxyribose (Halliwell et al., 1987). In the present study, a known scavenger of ^•^OH, mannitol and pirfenidone were added in increasing concentrations.

#### Patch Clamp Studies in Vascular Smooth Muscle

Vascular smooth muscle cells (VSMCs) were isolated by gentle trituration of rat small pulmonary arteries with a fire polished pippete after exposure to dissociation solutions containing papain first and secondly, collagenases H and F for 5 and 2 min, respectively, as previously described (Engholm et al., 2016). Freshly isolated cells were used for patch-clamp recordings within 5 h after the isolation procedure. Current-voltage relations were determined using 600 ms voltage ramps from −100 to 160 mV and a holding potential (Vh) of −65 mV for SMCs (Engholm et al., 2016). Amplitudes of K^+^-outward currents in control conditions and in response to were measured and normalized to the maximal amplitude of the current in control condition (I/I_max_ _Control_) in the absence and the presence of iberiotoxin and the Kv7 channel blocker XE991. Extracellular patch-clamp solution for VSMCs contained the following (mmol/L): 130 NaCl, 5 KCl, 1.2 MgCl_2_, 1.5 CaCl_2_, 10 glucose and 10 HEPES (adjusted to pH 7.3 with NaOH). The patch pipette (intracellular) solution for SMCs contained the following (mmol/L): 130 KCl, 1.2 MgCl2, 0.1 EGTA, and 10 HEPES (adjusted to pH 7.2 with KOH).

### Investigation of the Effect of Pirfenidone on Endothelial Function in the Aorta and Mesenteric Arteries From Diabetic Mice

#### Functional Studies in the Aorta and Mesenteric Small Arteries From Diabetic Animals

From 16–18-weeks control db/db+ and diabetic db/db animals, the descending thoracic aorta was dissected and aorta segments of 2 mm were mounted on two 100-µm wires, while mesenteric small arteries were mounted on 40-µm wires in microvascular myographs (Danish Myotechnology, Aarhus, Denmark) for isometric tension recordings. The arterial segments were stretched to their optimal diameter, which corresponded to an internal circumference of 90% of that achieved when the vessels were exposed to a passive tension yielding a transmural pressure of 100 mmHg. The baths were heated to 37 °C and equilibrated with 5% CO_2_ to maintain the desired pH of 7.4. The viability of the segments was tested by their ability to contract to potassium-rich PSS (KPSS, 60 mM) and to phenylephrine (10^−7^ M).

To investigate the effect of pirfenidone in aorta segments and mesenteric small arteries from db/db+ and db/db mice, concentration-response curves for pirfenidone were obtained in phenylephrine-contracted preparations. To investigate the effect of pirfenidone on endothelium-dependent relaxation, the preparations were incubated with pirfenidone (3 × 10^−5^ M) for 30 min, and in phenylephrine (10^−7^ M)-contracted preparations, concentration-response curves were obtained for acetylcholine and the NO donor, sodium nitroprusside (SNP).

Pirfenidone was found to increase acetylcholine relaxation in aorta from db/db mice, while this was not the case in mesenteric arteries. Thus, to investigate whether K_V_7 and BK_Ca_ channels are involved in the effect of pirfenidone on acetylcholine relaxation, aorta segments from db/db+ and db/db male mice were incubated with pirfenidone (3 × 10^−5^ M), an opener of K_V_7 channels, flupirtine (10^−5^ M), the combination of pirfenidone and flupirtine, a blocker of K_V_7 channels, XE991 (10^−5^ M), a blocker of BK_Ca_ channels, iberiotoxin (10^−7^ M), and a combination of pirfenidone with either XE991 or iberiotoxin. Then the preparations were contracted with phenylephrine and concentration-response curves were obtained for acetylcholine. Parallel control curves were performed in the presence of the vehicle.

#### Immunoblotting

Immunoblotting was performed for K_V_7.4, K_V_7.5, and BK_Ca_ alpha- and beta_1_-subunit in the aorta. Aorta segments were isolated from db/db+ and db/db mice and frozen at −80 °C. The primary investigator was blinded toward the samples. The tissue was homogenized in lysis buffer (20 mM tris/HCl, 5 mM EGTA, 150 mM NaCl, 20 mM glycerophosphate, 10 mM NaF, 1% Triton X-100, 0.1% tween-20, 1x Halt™ protease and phosphatase inhibitor cocktail) by using a pestle, then the sample was centrifuged 10 min at 10.000 rpm at 4 °C, and the supernatant frozen at −80 °C. Total protein was quantified using the Bio-Rad Protein Assay (Bio-Rad, Hercules, CA).

Equal amounts of total protein (5 µg) were resolved by electrophoresis on a 4–20% Criterion™ TGX Stain-Free™ SDS-polyacrylamide Gel (Bio-rad, Hercules, California) under reducing conditions. Before transfer, the gel was activated by UV-light for 5 min. After transfer, the PVDF- membrane (PerkinElmer, Waltham, Massachusetts) was reactivated by UV-light and the total amount of protein was visualized for loading control and normalization (Gilda and Gomes, 2013). The membranes were blocked overnight at 4 °C in 0.3% I-block, washed, and incubated with primary antibody: polyclonal anti-BKα (1:100; Alomone, APC-107, RRID: AB_2040091, Jerusalem, ISR) (Comerma-Steffensen et al., 2017), monoclonal anti-BKβ (1:250; Abcam, Ab3587, RRID:AB_303,932, Cambridge, United Kingdom), polyclonal anti-Kv7.4 (1:200; Santa Cruz Biotechnology, SC50417, RRID:AB_2131729 California, United States) (Hedegaard et al., 2014), polyclonal anti-Kv7.5 (1:100; Santa Cruz Biotechnology, SC50416, RRID:AB_2131834, California, United States) (Hedegaard et al., 2014), and panactin (1:1.000; Cell Signaling Technology Cat# 4968, RRID:AB_2313904, Massachusetts, United States) all diluted in 0.3% I-block. This was followed by incubation with a relevant horseradish-peroxidase (HRP) labeled secondary antibody (1:4.000) (Santa Cruz Biotechnologies, Santa Cruz, California, United States).

The blot was developed using Western LightningTM Chemiluminescence Reagent Plus (NEN-LifeTM Science Products). The intensity of the fluorescence in each band was measured with an image analyzer (PXi, Syngene, United Kingdom). The bands were normalized to total protein in the membrane and also expressed relative to the loading control pan-actin. Controls for K_V_7 channels and BK_Ca_ subunits were included as previously described (Hedegaard et al., 2014; Comerma-Steffensen et al., 2017).

#### Effect of Pirfenidone in Anesthetized Diabetic Mice

Mice were anesthetized using sodium pentobarbital (50 mg kg^−1^, i. p.) and then placed on a heating pad to maintain body temperature at 37 °C. Three electrodes were positioned in an Einthoven’s triangle configuration to obtain heart rate and electrical activity of the heart. Catheters were surgically implanted into the jugular vein for infusion of test drugs and in the carotid artery for measurement of blood pressure. All measurements were continuously recorded with a computerized data acquisition system (PowerLab, ADInstruments). The primary investigator was blinded toward the drugs. Before the start of the experimental protocol, blood pressure and heart rate were allowed to stabilize for 10 min. Using syringe pumps (Havard Apparatus), acetylcholine (0.001 mg kg^−1^) was then infused followed by infusion of increasing doses of pirfenidone (0.1, 1.0, and 10 mg kg^−1^) and final re-administration of acetylcholine (0.001 mg kg^−1^). Each drug infusion lasted 3 min and was separated by a 5-min stabilization period. At the end of the experiment, mice were sacrificed for the extraction of tissue samples using cervical dislocation.

### Materials

The following drugs were used: Acetylcholine, phenylephrine hydrochloride (PE), iberiotoxin, SNP (sodium nitroprusside dehydrate), U46619 (9α-epoxymethanoprostaglandin F_2α_), pirfenidone (5-methyl-1-phenyl-2-(1H)-pyridone), flupiritine (ethyl 2-amino-6-((p-fluorobenzyl)amino)-3-pyridinecarbamate), mannitol, N^G^-nitro-l-arginine (L-NOARG), tempol, TEA (tetraethylammonium), TRAM-34, UCL1684, and XE991 (10,10-bis(4-pyridinylmethyl)-9(10H)-anthracenone) were purchased from Sigma (St Louis, MO, United States). TRAM-34, UCL1684, and XE991 were dissolved in dimethylsulphoxide (DMSO), and further diluted in distilled water. Unless otherwise stated the substances were dissolved in distilled water. The concentration of DMSO in the bath was below 0.01% and to examine whether vehicle affected vascular contractility parallel control curves for the vehicle were obtained.

#### Data and Statistical Analysis

The data and statistical analysis comply with the recommendations on experimental design and analysis in pharmacology (Curtis et al., 2018). The data are presented as means ± standard error of the mean. Statistical analyses were performed using Stata13 (StataCorp, Texas, United States) or GraphPad Prism 7.02 (GraphPad Software Inc., RRID:SCR_002798, California, United States). Differences between the db/db+ and db/db mice were compared by the unpaired Student’s t-test, Welch’s *t*-test for unequal variances for parametric data or Mann-Whitney test for non-paramteric data. Concentration-response curves were constructed using a nonlinear curve-fit model and the half-maximal relaxation (EC_50_) was determined:

$$y=\text{bottom}+\frac{\left(\text{top}-\text{bottom}\right)}{1+10^{\left(\left(\log EC50-x\right)*\text{slope}\right)}}$$

Two-way ANOVA was used to test for differences in concentration-response curves in isolated vessel segments. Concerning in infusion of acetylcholine and pirfenidone *in vivo*, the responses were compared to baseline using a Wilcoxon test. The assumptions of parametric data to the ANOVA approach were verified by inspection of Q-Q plots. The significance level was *p < 0.05 for all tests.

## Results

### Mechanisms Involved in Pirfenidone Relaxation

#### Pirfenidone-Induced Relaxation in Rat Pulmonary Arteries

**FIGURE 1:**
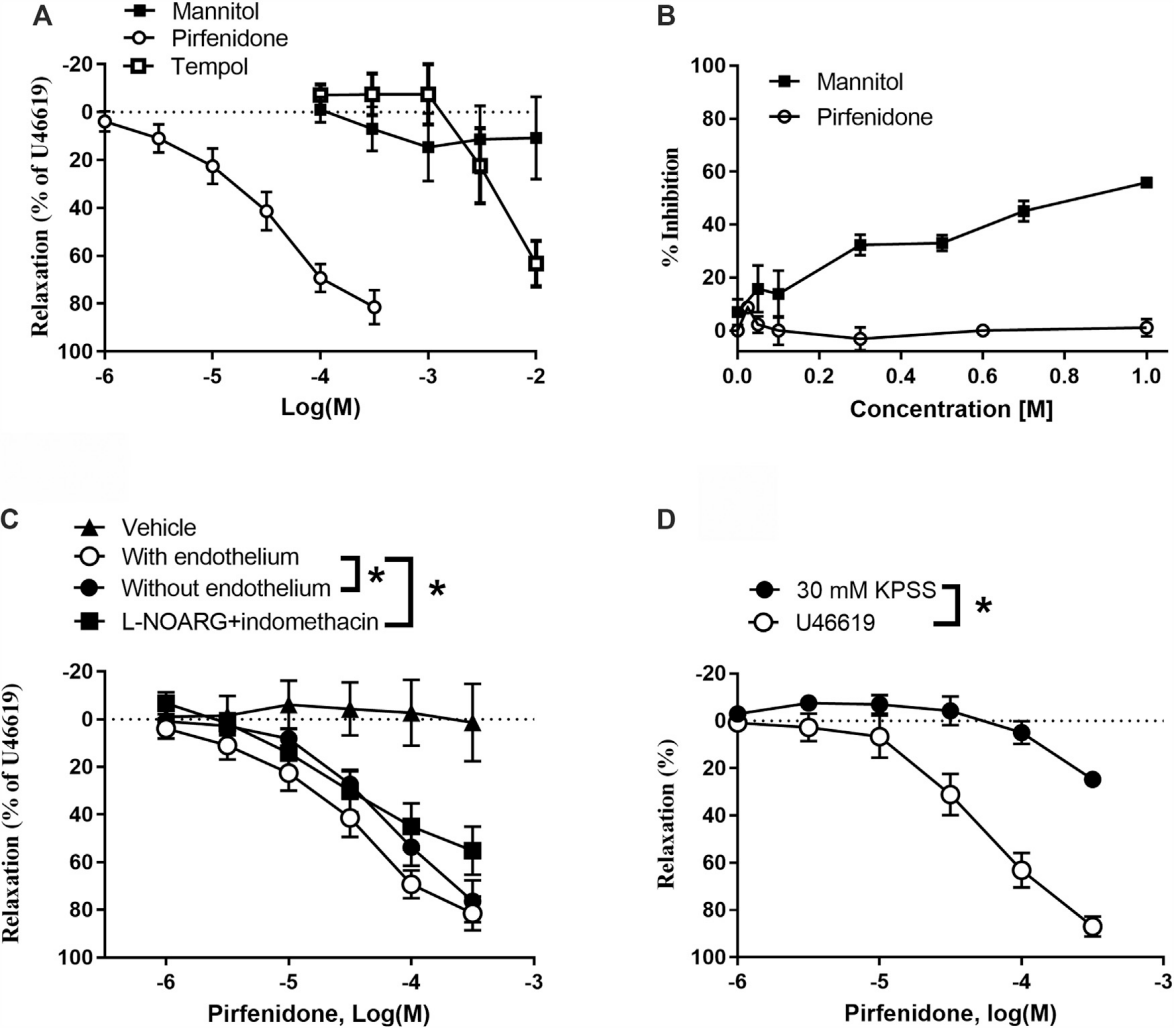
Direct vasodilator effect of pirfenidone in rat pulmonary arteries involve the endothelium and potassium channels. **(A)** In U44619-contracted rat pulmonary arteries, pirfenidone (*n* = 10) induced concentration-dependent relaxations. Mannitol (*n* = 5) failed to relax the preparations, while tempol (*n* = 5) only induced relaxations at very high concentrations. **(B)** Inhibition of the degradation of deoxyribose by ^•^OH by the hydroxyl scavenger D-mannitol by up to 60% inhibition, while there is no effect of pirfenidone on the degradation of deoxyribose by ^•^OH (averages of three independent experiments). **(C)** In U46619 (3 × 10^−8^ M) contracted arteries, pirfenidone (*n* = 8) induced relaxations, which were reduced in the presence of an inhibitor of nitric oxide synthase, L-NOARG, and of cyclooxygenase (3 × 10^−6^ M) (*n* = 8). In arteries without endothelium (*n* = 6), pirfenidone relaxation was markedly reduced. Vehicle did not affect vascular tone (*n* = 6). **(D)** In preparations contracted with 30 mM KPSS, prefenidone relaxations were markedly reduced compared to effect in U46619-contracted preparations. The results are means ± s. e.mean, where n indicates the number of animals examined. *p < 0.05 vs. pirfenidone control curves, two-way ANOVA.

Vasodilatation could be a contributor to the antifibrotic effects of pirfenidone in the lungs. To explore this potential effect, we studied the direct effect of pirfenidone on vascular tone in pulmonary arteries. In rat pulmonary arteries contracted with U46619, pirfenidone induced relaxations with EC_50_ values of 3.8 ± 1.4 × 10^−5^ M (*n* = 10) with maximum relaxations of 81 ± 4% (*n* = 8), while increasing concentrations (10^−4^-3 × 10^−2^ M) of mannitol failed to relax pulmonary arteries and only high concentrations (>10^−4^ M) of tempol induced relaxations (Figure 1A). Hydroxyl (^•^OH) scavenger studies showed that mannitol inhibited concentration-dependently the formation of pink chromogen confirming the ability of the compound to scavenge ^•^OH (Figure 1B). In contrast, pirfenidone failed to inhibit the formation of pink chromogen suggesting that at therapeutic concentrations pirfenidone does not appear to scavenge ^•^OH (Figure 1B).

Pirfenidone relaxations were significantly decreased by endothelial cell removal or by incubation with L-NOARG plus indomethacin (Figure 1C), while in preparations contracted with 60 mM KPSS, pirfenidone did not induce relaxation (Figure 1D).

#### Mechanisms Involved in Pirfenidone Relaxation in Mouse Coronary Arteries

**FIGURE 2:**
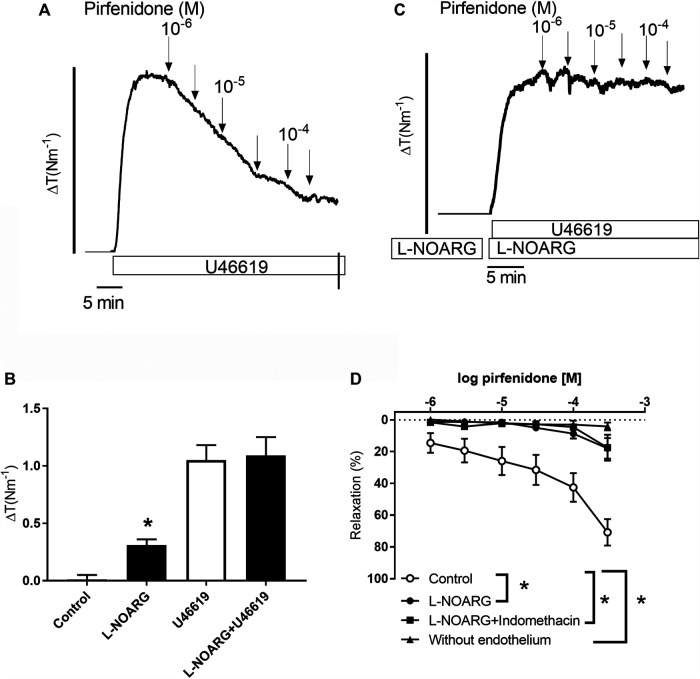
Pirfenidone induces endothelium-dependent nitric oxide-mediated relaxations in mouse coronary arteries. **(A)** Original trace showing contraction to the thromboxane analogue, U46619 and relaxations to increasing concentrations of the antifibrotic drug, pirfenidone. Horizontal bar shows time and vertical bar increase in tension (ΔT, 2 Nm^−1^). **(B)** Average increases in tension (ΔT) in control conditions, after incubation with an inhibitor of nitric oxide synthase L-NOARG (10^−3^ M), addition of U46619 (10^−7^ M), and L-NOARG plus U46619. **(C)** Original trace showing pirfenidone relaxations are abolished in coronary artery contracted with U46619 in the presence of L-NOARG. **(D)** Average relaxations induced by pirfenidone in the absence (*n* = 10) and the presence of L-NOARG (*n* = 7), and in the presence of L-NOARG plus indomethacin (3 × 10^−6^ M, *n* = 6), an inhibitor of cyclooxygenase. The results are means ± s.e. mean, where n indicates the number of animals examined. *p < 0.05 versus pirfenidone control curves, two-way ANOVA.

Pirfenidone has recently been shown to have cardioprotective effects, mainly through the inhibition of fibrosis, however, a direct vasodilatatory effect could also contribute to this effect. We investigated coronary arteries with internal lumen diameters of 195 ± 39 µm (*n* = 54) from wild type mice to examine the mechanisms involved in pirfenidone relaxation. In arteries contracted with the thromboxane analogue U46619, pirfenidone induced concentration-dependent relaxations which at 10^−4^ M pirfenidone were 71 ± 8% (*n* = 10) (Figure 2). Incubation with an inhibitor of NO synthase, L-NOARG (10^−3^ M) increased basal tension significantly suggesting inhibition of basal NO release, while the magnitude of U46619 contraction was similar in the absence and presence of L-NOARG by lowering the concentration of U46619 (Figure 2B). Relaxations induced by 10^−4^ M pirfenidone were reduced to 18 ± 6% (*p* < 0.05, *n* = 7) in the presence of, L-NOARG suggesting NO is involved in pirfenidone relaxation (Figure 2C). There was no additional effect of incubation with L-NOARG, and an inhibitor of cyclooxygenase, indomethacin (Figure 2D). Acetylcholine (10^−5^ M) relaxed mouse coronary arteries with endothelium 72 ± 6% (*n* = 10), but acetylcholine (−3 ± 2%, *n* = 6) and pirfenidone did not change vascular tone in preparations without endothelium (*n* = 6) (Figure 2D).

**FIGURE 3:**
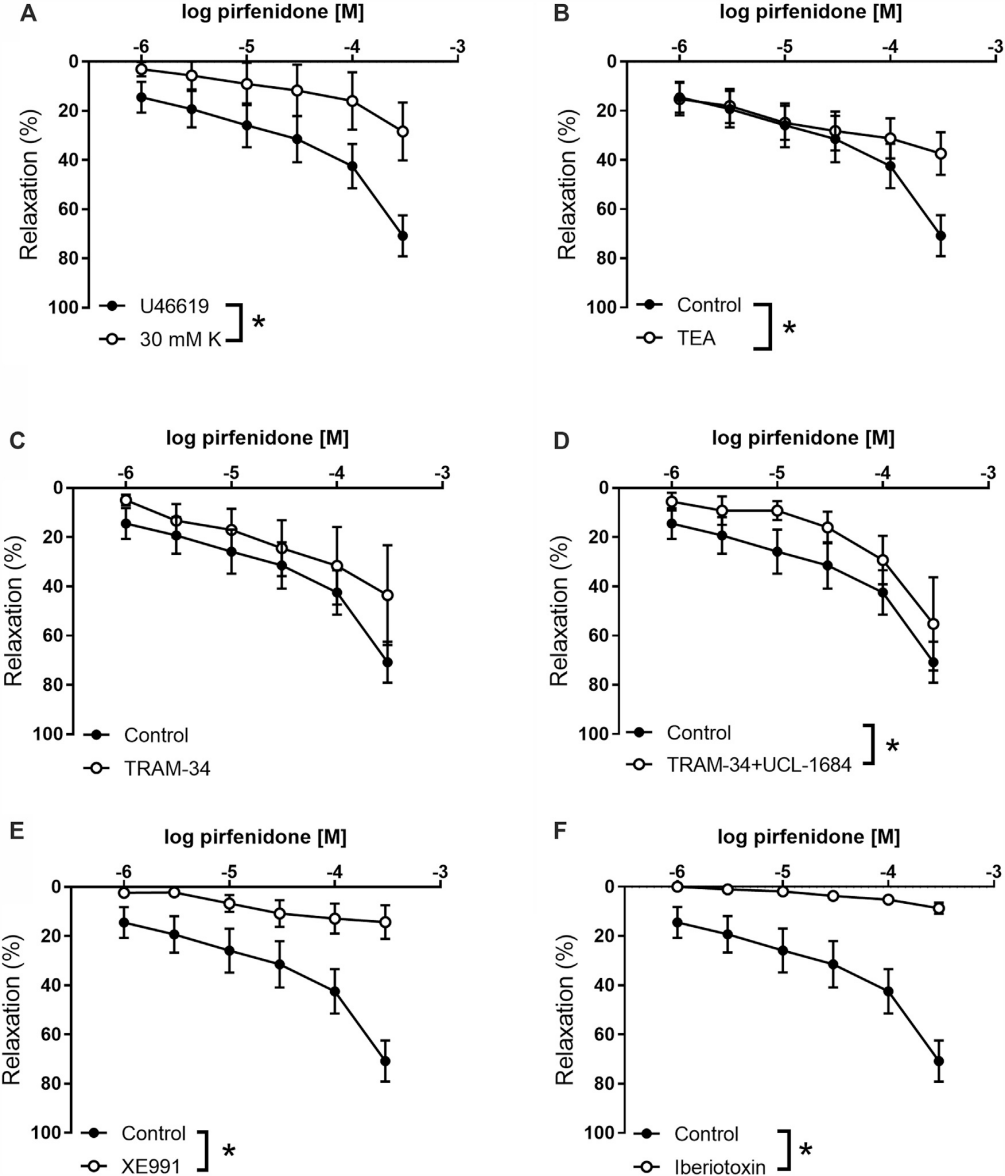
Involvement of potassium channels in pirfenidone relaxation of mouse coronary arteries. **(A)** Average relaxations induced by increasing concentrations of pirfenidone in preparations contracted by, respectively, U46619 (*n* = 10) and high extracellular potassium (30 mM, *n* = 7). Concentration-response curves were performed for pirfenidone in U46619-contracted arteries in the absence the presence of **(B)** a blocker of calcium-activated K channels, tetraethylammonium (1 mM TEA, *n* = 6), **(C)** TRAM-34 (10^−6^ M, *n* = 5), a blocker of intermediate-conductance calcium-activated potassium channels, **(D)** TRAM-34 and UCL1684 (10^−6^ M, *n* = 5) a blocker of small-conductance calcium-activated potassium channels, **(E)** a blocker of voltage-gated potassium channels of the K_V_7 subtype, XE0991 (10^−5^ M, *n* = 6), and **(F)** a selective blocker of large-conductance calcium-activated K channels, iberiotoxin (10^−8^ M, *n* = 6). The results are means ± s. e.mean, where n indicates the number of animals examined. *p < 0.05 vs. pirfenidone control curves, two-way ANOVA.

To investigate the role of potassium channels in pirfenidone relaxation, coronary small arteries were contracted with U46619 or 30 mM KPSS. Pirfenidone relaxations were significantly decreased in 30 mM KPSS vs. U46619-contracted coronary arteries (Figure 3A). Thus, the response to 10^−4^ M pirfenidone was reduced to 28 ± 12% (*p* < 0.05, *n* = 6) in the presence of high extracellular potassium (30 mM), which indicated an important involvement of potassium channels. To examine the subtype of potassium channels involved in the pirfenidone relaxations, the preparations were incubated with different potassium channel blockers. TEA (10^−3^ M) which blocks BK_Ca_ channels along with some types of voltage-gated potassium channels (i.e., K_V_1) (Nelson and Quayle, 1995), decreased pirfenidone relaxations (Figure 3B). TRAM-34, a blocker of IK_Ca_ channels failed to inhibit pirfenidone relaxation (Figure 3C). However, the combination of TRAM-34 with UCL1684, a blocker of SK_Ca_ channels, decreased pirfenidone relaxation in mouse coronary arteries (Figure 3D). As these channels are involved in release of endothelium-derived NO (Stankevicius et al., 2011; Simonsen et al., 2019), these results suggest that pirfenidone modifies the release or the effect of NO in the smooth muscle layer. A blocker of voltage-gated K_V_7 channels, XE991 (10^−5^ M) blocked the pirfenidone relaxation (Figure 3E), and 10^−4^ M pirfenidone relaxed the mouse coronary arteries with 14 ± 7% (*p* < 0.05, *n* = 6) in the presence of XE991. Incubation with a blocker of BK_Ca_ channels, iberiotoxin (10^−7^ M) markedly decreased pirfenidone relaxation (Figure 3F), and the response to 10^−4^ M pirfenidone was 9 ± 6% (*p* < 0.05, *n* = 6) in the presence of iberiotoxin. The BK_Ca_ and K_V_7 channels are mainly expressed in the smooth muscle layer, and therefore these results suggest that pirfenidone enhance the effect of NO in the smooth muscle layer by modulating BK_Ca_ or K_V_7 channel. This is further supported by the observation that pirfenidone significantly improved relaxations induced by the NO donor, SNP in coronary arteries from diabetic male mice (Supplementary Figure S1).

#### Patch Clamp Studies in Vascular Smooth Muscle Cells

**FIGURE 4:**
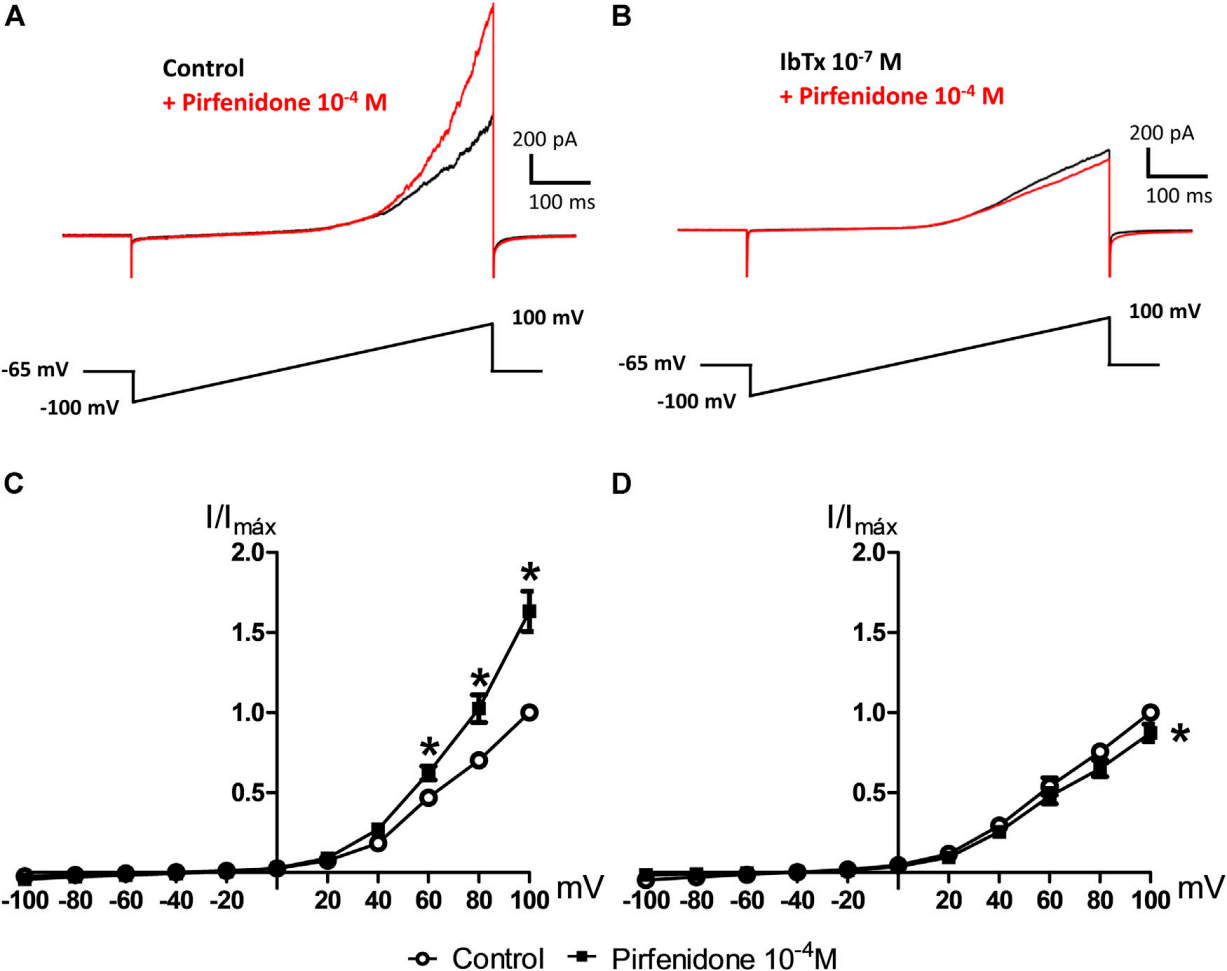
Effect of iberiotoxin on pirfenidone-induced current in vascular smooth muscle. **(A)** Original traces of whole-cell patch clamp showing current induced by voltage ramps before (control) and after the addition of pirfenidone (10^−4^ M). **(B)** Original traces showing the current increases in the presence of iberiotoxin without (control) and with pirfenidone. **(C)** Average current-voltage (I-V) relationships of isolated pulmonary vascular smooth muscle cells before and after short incubation with pirfenidone (10^−4^ M) in control conditions (*n* = 7) and **(D)** in the presence of iberiotoxin (10^−7^ M) (*n* = 7), and posterior addition of XE991 (10^−5^ M, *n* = 3). I-V relationship were determined using 600 ms voltage ramps (−100 to 100 mV; holding voltage, V_h_ = −65 mV). The results are means ± s. e.mean, where n indicates the number of animals examined. *p < 0.05 vs. current in the corresponding control conditions, two-way ANOVA. #*p* < 0.05 vs. currents in the presence of iberiotoxin after the addition of pirfenidone.

To study the role of potassium channels in the effects of pirfenidone in the pulmonary circulation, we performed patch-clamp studies. In whole-cell patch clamp I-V relationship were determined using 600 ms voltage ramps (−100 to 100 mV; holding potential Vh = −65 mV). In control conditions, pirfenidone (10^−4^ M) increased current (Figure 4A), an effect that was blocked in the presence of the BK_Ca_ channel blocker, iberitoxin (10^−7^ M) (Figure 4B). The average current induced by pirfenidone was significantly increased, and abolished by iberiotoxin (Figures 4C,D).

### Effect of Pirfenidone in the Aorta and Mesenteric Arteries From Diabetic Db/Db Mouse

Normoglycaemic db/db+ control mice weighed 30 ± 1 g (*n* = 24) and had blood glucose levels of 8 ± 1 mM (*n* = 24). Compared to control mice, weight (50 ± 2 g, *p* < 0.05, *n* = 24) and blood glucose levels (27 ± 2 mM, *p* < 0.05, *n* = 24) were higher in diabetic (db/db) male mice In female db/db mice, weight (51 ± 1 g, *p* < 0.05, *n* = 14) and blood glucose levels (29 ± 2 mM, *p* < 0.05, *n* = 14) were also higher compared to normoglycaemic db/db+ control mice that weighed 22 ± 1 g (*n* = 14) and had blood glucose levels of 6 ± 1 mM (*n* = 14).

**FIGURE 5:**
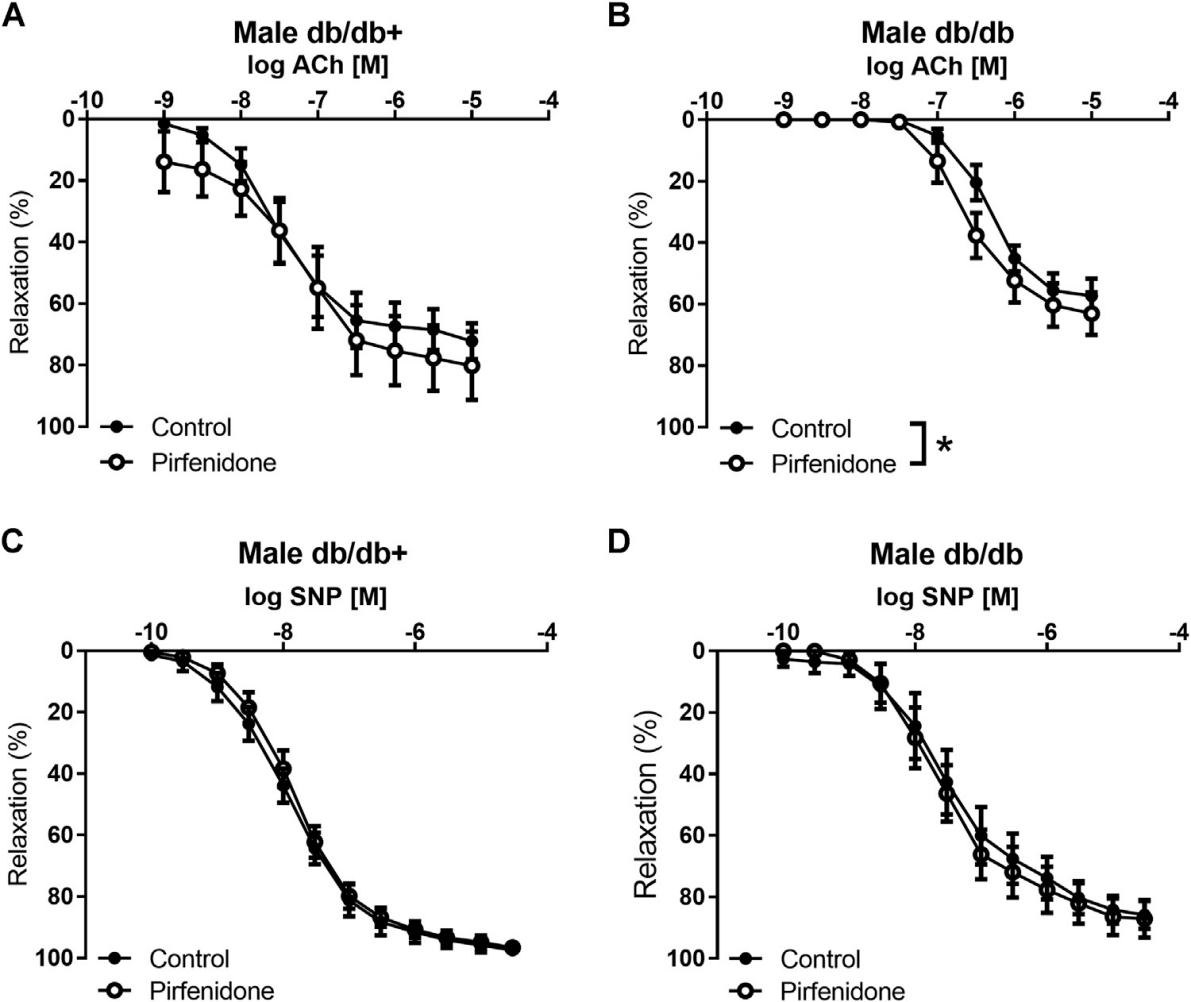
Pirfenidone enhances acetylcholine relaxation in aorta from male diabetic mice. Average relaxations induced by the endothelium-dependent vasodilator, acetylcholine in the absence and the presence of pirfenidone in phenylephrine-contracted aorta segments arteries from **(A)** heterozygous control (db/db+) (*n* = 6) and **(B)** diabetic (db/db) mice (*n* = 6). Average relaxations induced by the nitric oxide donor, sodium nitroprusside (SNP) in the absence and the presence of pirfenidone in phenylephrine-contracted aorta segments arteries from **(C)** db/db+ (*n* = 8) and **(D)** db/db mice (*n* = 6). The results are means ± s. e.mean, where n indicates the number of animals examined. *p < 0.05 vs. respective control curves, two-way ANOVA.

In aorta preparations from db/db+ mice contracted with phenylephrine, pirfenidone (10^−6^ to 3 × 10^−4^ M) failed to induce relaxatioin (*n* = 6) (data not shown). In phenylephrine-contracted aorta preparations, acetylcholine induced concentration-dependent relaxations with a maximum relaxation of 75.4 ± 5.8% (*n* = 7) in aorta from male db/db + mice, while maximum acetylcholine relaxations were reduced to 57.3 ± 5.5% (*p* < 0.05, *n* = 6) in aorta from male db/db mice (Figures 5A,B). The NO donor, SNP, induced concentration-dependent relaxations in aorta from male mice and these were unaltered when comparing the responses in db/db + and db/db mice (Figures 5C,D). In aorta from female mice, acetylcholine-induced relaxations were decreased in aorta from db/db vs. db/db+ mice, while SNP-induced relaxations were similar (Supplementary Figure S2). Incubation with pirfenidone (3 × 10^−5^ M) increased acetylcholine relaxation in aortas from both female and male db/db mice, while acetylcholine relaxation in the absence and the presence of pirfenidone was similar in aortas from db/db+ mice (Figures 5A,B; Supplementary Figures S2A,B). Pirfenidone (3 × 10^−5^ M) did not increase SNP relaxations in aortas from female and male mice (Figures 5C,D; Supplementary Figures S2C,D). These findings suggest that pirfenidone selectively enhances relaxations induced by the endothelium-dependent vasodilator, acetylcholine, in aorta from diabetic female and male db/db mice.

Acetylcholine and SNP relaxations were also impaired in mesenteric arteries from male db/db mice. Incubating with pirfenidone had no effect on acetylcholine relaxation or on SNP relaxation in mesenteric arteries from male diabetic db/db mice or normoglycaemic db/db+ control mice (Supplementary Figures S3A–D). Incubation with L-NOARG and indomethacin inhibited acetylcholine relaxation in the mesenteric arteries from db/db+ and db/db mice. In the presence of L-NOARG and indomethacin, pirfenidone had no effect on the reduced relaxations in mesenteric arteries of db/db mice (Supplementary Figures S2E,F).

### Mechanisms Involved in the Effect of Pirfenidone on Acetylcholine Relaxation in the Aorta From Diabetic Mice

**FIGURE 6:**
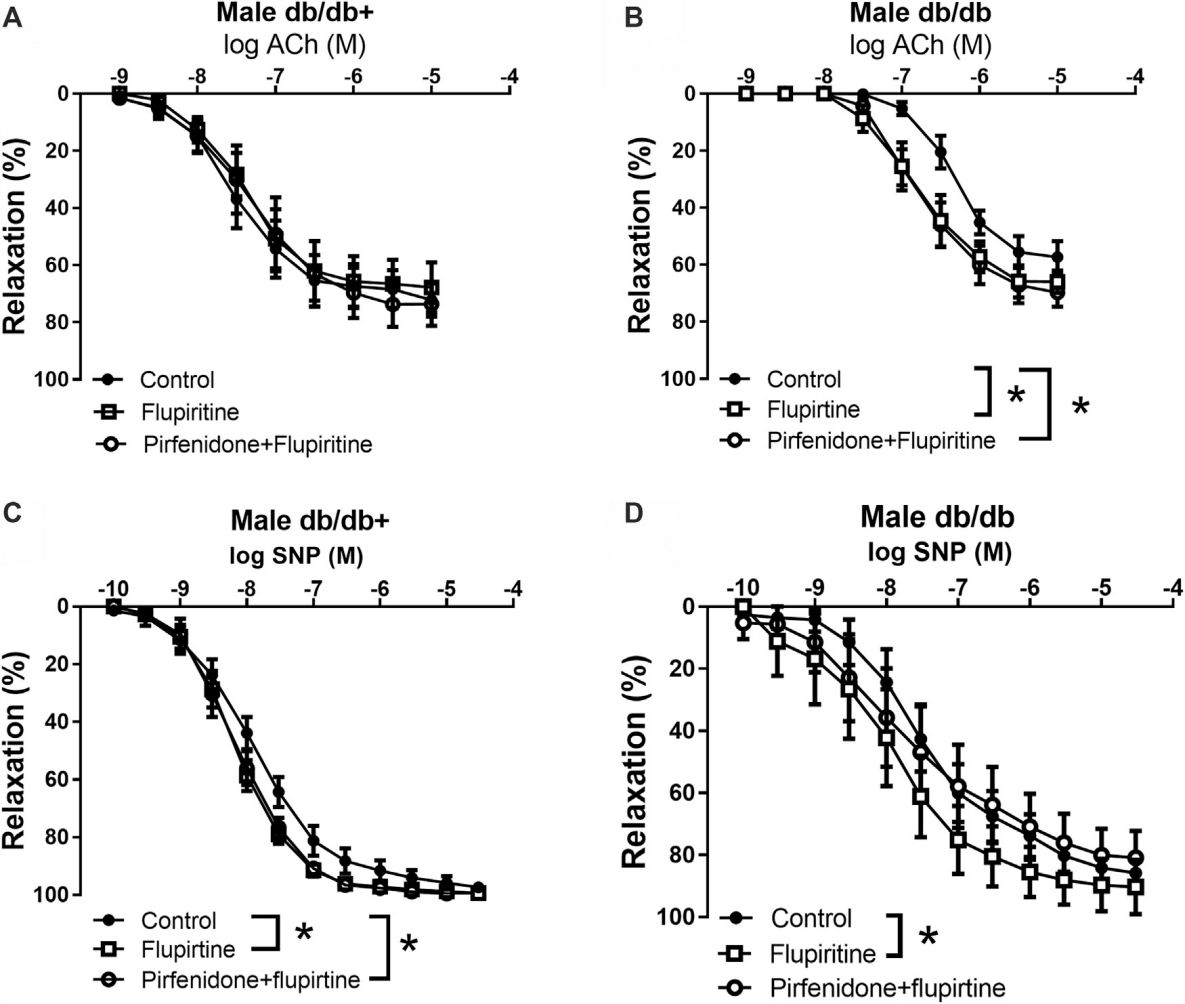
Effect of a K_V_7 channel opener on acetylcholine relaxation in aorta from male diabetic animals. Average relaxations induced by **(A, B)** acetylcholine (ACh) and **(C, D)** sodium nitroprusside (SNP) in the absence and the presence of the K_V_7 channel opener flupirtine (10^−6^ M) or flupirtine plus pirfenidone (3 × 10^−5^ M) in **(A, C)** heterozygous db/db + mice (*n* = 6), and in **(B, D)** diabetic db/db mice (*n* = 6). The results are means ± s. e.mean, where n indicates the number of animals examined. *p < 0.05 vs. respective control curves, two-way ANOVA.

**FIGURE 7:**
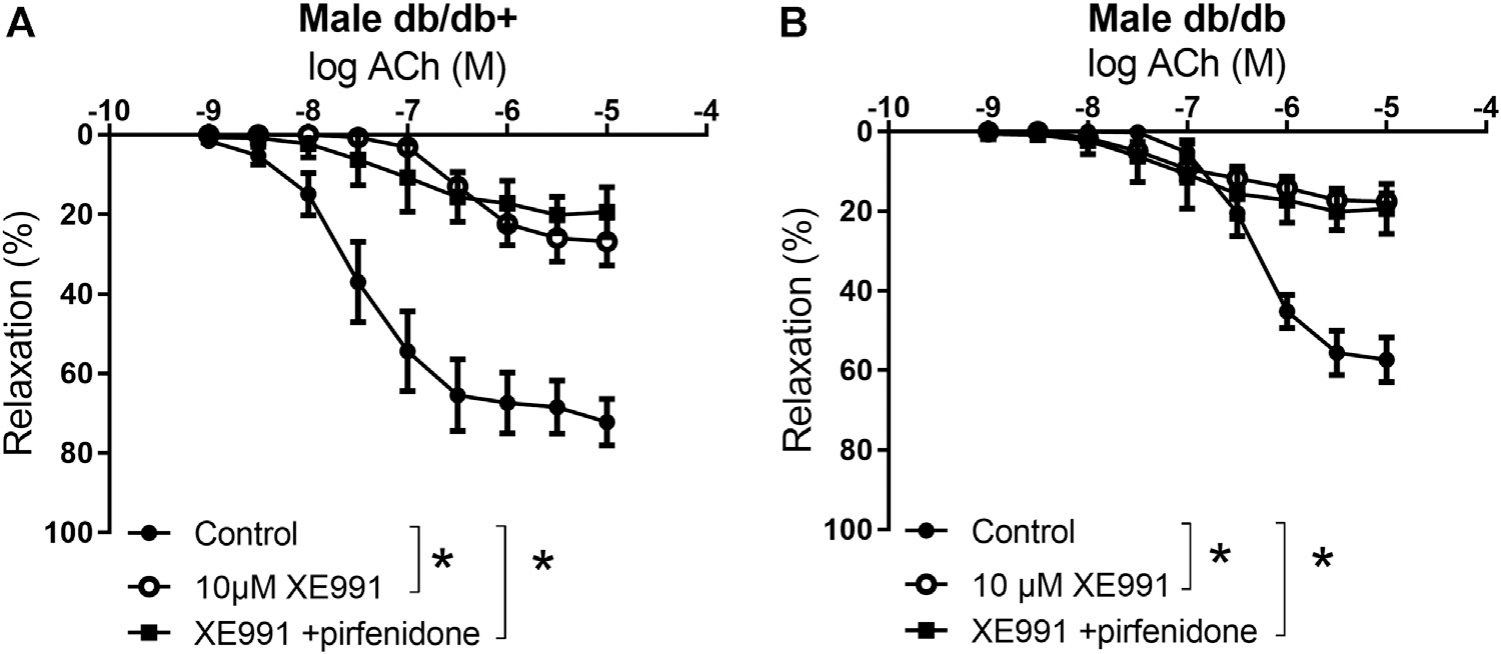
The K_V_7 channel blocker XE991 inhibits the potentiating effect of pirfenidone on acetylcholine relaxation in aorta from diabetic animals. Average relaxations induced by acetylcholine in the absence and absence of K_V_7 channel blocker, XE0991 (10^−5^ M), or XE0991 combined with pirfenidone in **(A)** db/db+ mice (*n* = 6) and **(B)** diabetic db/db mice (*n* = 5). The results are means ± s. e.mean, where n indicates the number of animals examined. *p < 0.05 vs. respective control curves, two-way ANOVA.

**FIGURE 8:**
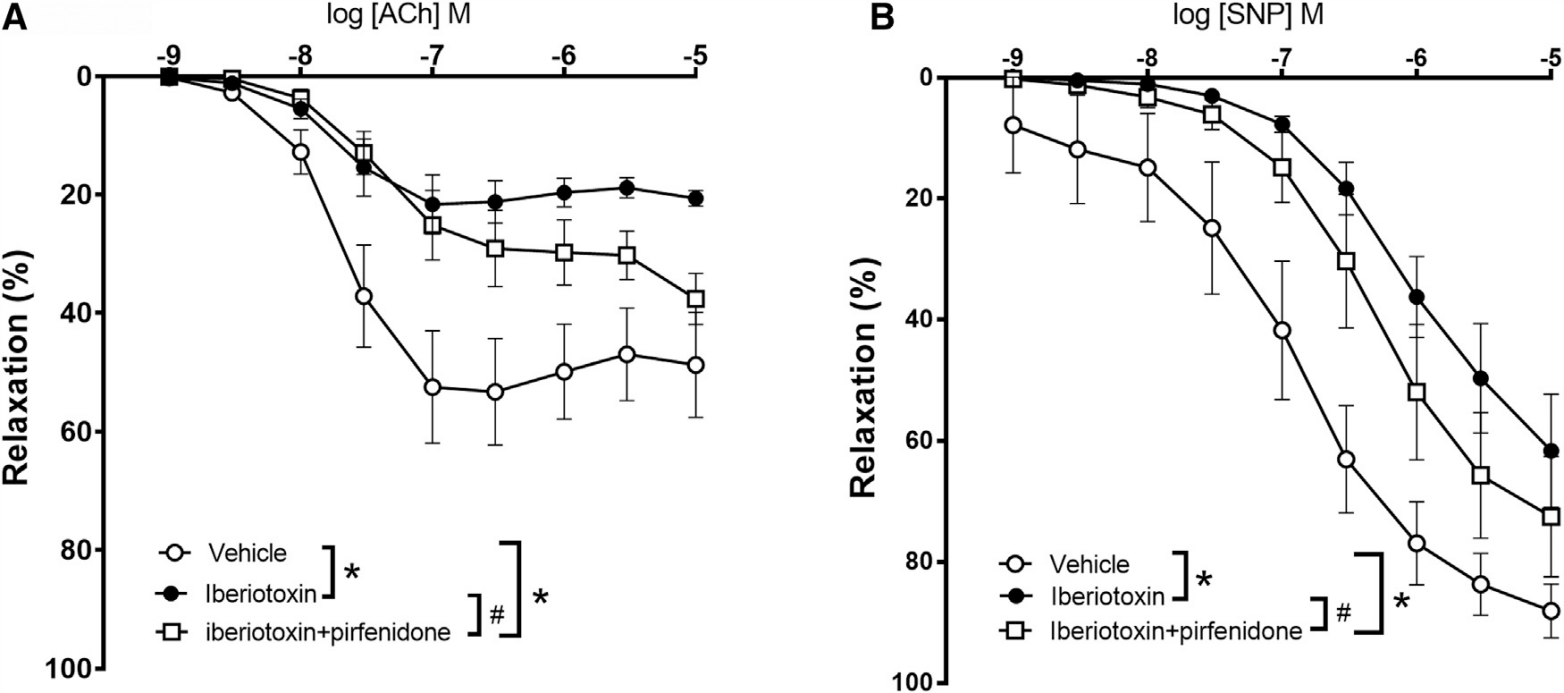
Effect of iberiotoxin, a blocker on large-conductance calcium-activated potassium channels (BK_Ca_) on acetylcholine and sodium nitroprusside relaxations in aorta from diabetic (db/db) mice. Average relaxations induced by **(A)** acetylcholine in control conditions (*n* = 6) and in the presence of iberiotoxin with (*n* = 5) and without pirfenidone (*n* = 6) and by **(B)** sodium nitroprusside (SNP) in control conditions (*n* = 6) and in the presence of iberiotoxin with (*n* = 5) and without pirfenodine (*n* = 6). *p < 0.05 vs. respective control curves; ^#^ *p* < 0.05 vs. concentration-response curve in the presence of iberiotoxin, two-way ANOVA.

To investigate the mechanisms involved in the effect of pirfenidone on acetylcholine relaxation in the aorta, aortas from male mice were incubated with an opener of K_V_7 channels, flupirtine (10^−5^ M) and relaxation curves for acetylcholine were obtained. Flupirtine increased acetylcholine relaxation in aortas from db/db male mice, while there was no effect on acetylcholine relaxation in aorta from normoglycemic db/db+ mice (Figures 6A,B). The combination of flupirtine and pirfenidone did not cause additional effect compared to each of the drugs (Figure 6B). Flupirtine increased SNP relaxations in the aortas from both db/db+ and db/db mice, while there was no additive effect on SNP relaxation by combining flupirtine and pirfenidone (Figures 6C,D). Moreover, a blocker of K_V_7 channels, XE991 (10^−5^ M), markedly inhibited acetylcholine relaxation in aorta from both db/db+ and db/db male mice (Figures 7A,B). In the presence of XE991, pirfenidone (3 × 10^−5^ M) did not increase acetylcholine relaxation in aortas from db/db mice (Figure 7B). To explore the role of BK_Ca_ channels in the effect of pirfenidone, aortas were incubated with iberiotoxin that decreased acetylcholine relaxation markedly in aortas from male db/db mice (Figure 8A), but pirfenidone increased acetylcholine relaxation also in the presence of iberiotoxin. Iberiotoxin decreased relaxations induced by the NO donor, SNP, and pirfenidone increased SNP relaxations in the presence of iberiotoxin (Figure 8B). These findings suggest that pirfenidone increases acetylcholine relaxation by a mechanism involving smooth muscle channels sensitive to K_V_7 channel modulating drugs.

### Expression of K_V_7 and BK_Ca_ Channels in the Aorta of Diabetic Mice

**FIGURE 9:**
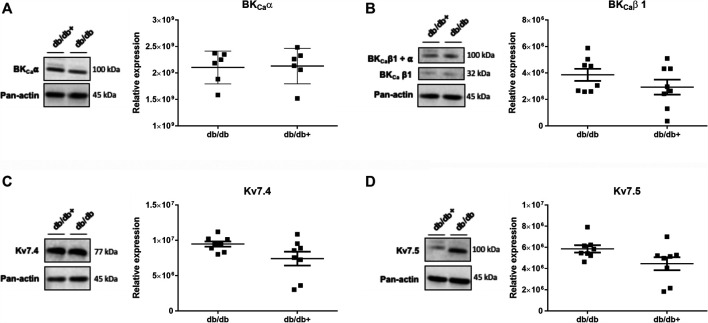
Immunoblotting for subunits of the large-conductance calcium-activated potassium channels (BK_Ca_α and BK_Ca_β) and for voltage-gated K_V_7.4 and K_V_7.5 channels in the aorta of male diabetic db/db mice. The Figures show inserts of original immunoblottings and average values expressed as a ratio to the loading control pan-actin in aorta from heterozygote db/db + control mice (*n* = 8) and diabetic db/db (*n* = 8) mice. **(A)** BK_Ca_α (*n* = 6 from each group), **(B)** BK_Ca_β1 (*n* = 8 from each group), **(C)** K_V_7.4 (*n* = 7 from each group), and **(D)** K_V_7.5 (*n* = 7 from each group). The results are means ± s. e. mean from. There were no significant differences, Student’s t-test.

To investigate whether the effect of pirfenidone in aorta from male db/db mice is due to changes in expression of K_V_7 and/or BK_Ca_ channels, immunoblotting was performed. The quantification of the blots revealed that expressions of BK_Ca_ channel pore α subunit and the auxiliary β subunit were unaltered in aorta from db/db vs. db/db+ mice (Figures 9A,B). The expression of K_V_7.4 was more pronounced than that of K_V_7.5, but there were no significant differences in expression in aorta from diabetic db/db compared to aorta from db/db+ mice (Figures 9C,D), although there was a tendency for K_V_7.5 channel expression to be upregulated (*p* = 0.06). The protein expression is expressed relative to panactin. Expression relative to total protein did not change the results, but tendencies were still the same (Supplementary Figure S4).

### Effect of Pirfenidone on Heart Rate and Blood Pressure in Anesthetized Diabetic Mice

**FIGURE 10:**
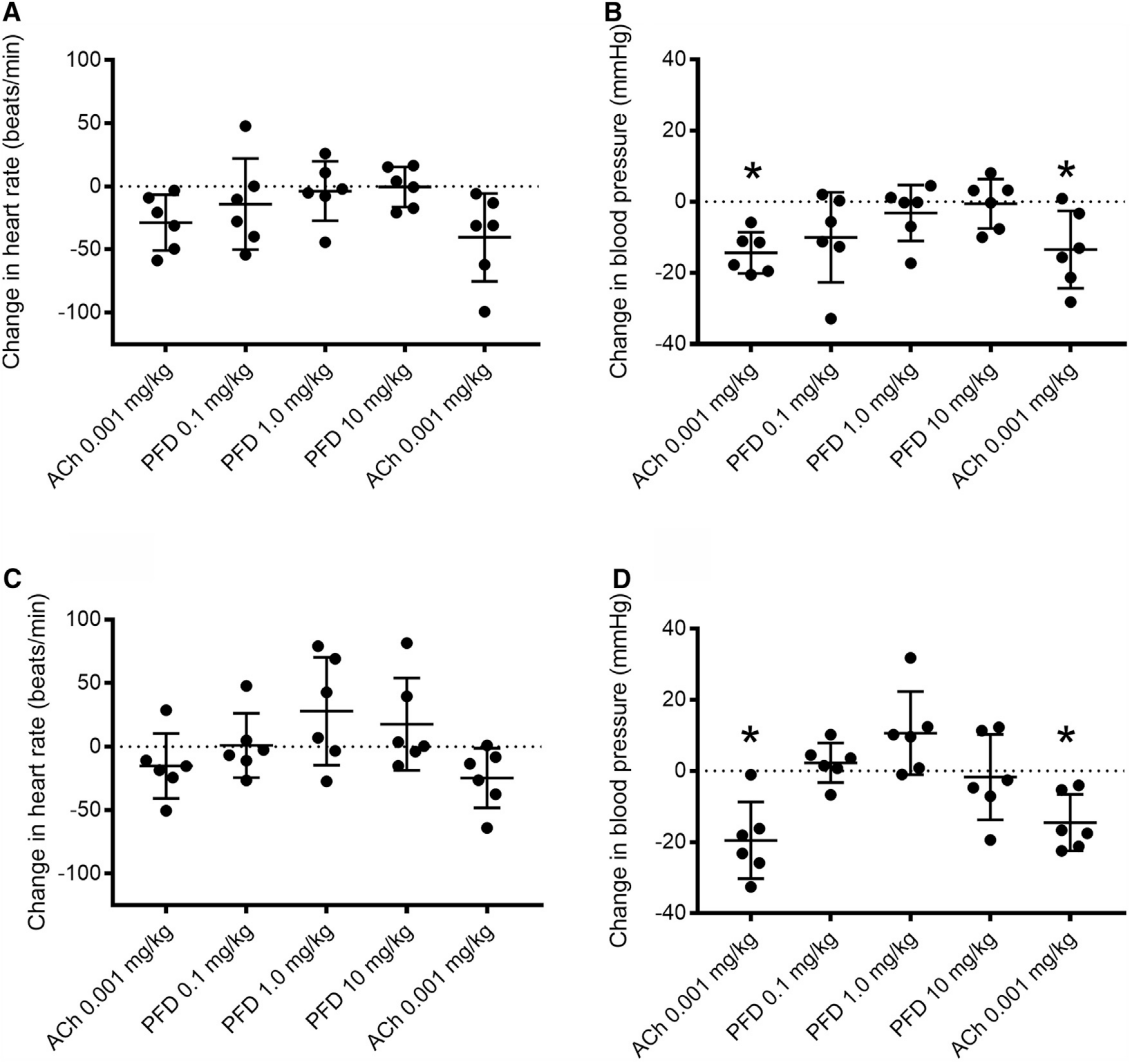
Effect of pirfenidone alone and in combination with acetylcholine on heart rate and mean arterial blood pressure in anesthetized db/db+ and diabetic (db/db) mice. Acetylcholine (ACh) infusion decreased mean arterial blood pressure and heart rate, while pirfenidone (PFD) by itself failed to change heart rate and blood pressure in anesthetized **(A, B)** db/db+ and **(C, D)** db/db mice. The response to acetylcholine was unaltered in the presence of the pirfenidone. The results are means ± s. e. mean, where n indicates the number of animals examined. *p < 0.05 compared to baseline, Wilcoxon test.

To investigate the acute effect of pirfenidone on acetylcholine-induced changes in mean arterial blood pressure (MAP), acetylcholine was infused followed by incremental doses of pirfenidone and a final acetylcholine infusion. Pirfenidone acutely did not decrease heart rate or MAP in diabetic db/db and db/db+ mice (Figure 10). In male db/db+ and diabetic db/db mice, infusion of acetylcholine significantly decreased MAP before infusion of pirfenidone, and similar decrease in blood pressure was also observed to acetylcholine infusion after testing the highest dose of pirfenidone (Figures 10B,D).

## Discussion

**FIGURE 11:**
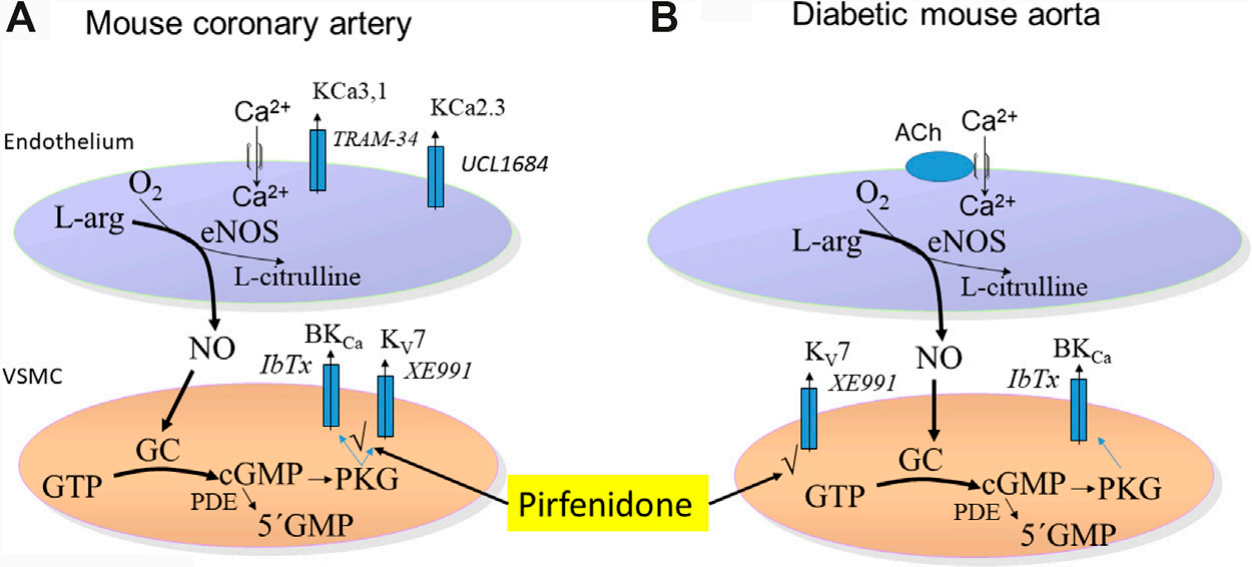
Schematic presentation of the mechanism involved in the vasodilator effects of pirfenidone. **(A)** Mechanisms involved in pirfenidone-induced vasodilatation in mouse coronary arteries involve pirfenidone through targeting upstream pathway (v) leading to opening of BK_Ca_ and K_V_7 channels increase the relaxations induced by basally-released nitric oxide (NO). **(B)** In the aorta from diabetic mice, acetylcholine relaxations are potentiated by pirfenidone supporting opening of K_V_7.4 channels. There is no effect of pirfenidone in the aorta from normoglycaemic animals.

We investigated the effect of the antifibrotic drug, pirfenidone, on vascular tone. In mouse coronary arteries, pirfenidone induced concentration-dependent relaxations, which were inhibited by an inhibitor of NO synthase, high extracellular potassium, and blockers of BK_Ca_ and K_V_7 channels suggesting an involvement of NO and of these channels in pirfenidone relaxations (Figure 11A). These findings were further supported by the observation that pirfenidone relaxed rat pulmonary arteries and increased iberiotoxin-sensitive currents in isolated vascular smooth muscle cells. In the aorta from diabetic mice, pirfenidone and the K_V_7 channel opener, flupirtine, restored the impaired acetylcholine relaxations in aorta from type 2 diabetic db/db mice. The K_V_7 channel blocker XE991 decreased acetylcholine relaxations, and in contrast to the BK_Ca_ blocker iberiotoxin, blocked the effect of pirfenidone on acetylcholine relaxations in the aorta from diabetic mice. Together with the observations that BK_Ca_ channels subunits, K_V_7.4, and K_V_7.5 channels are expressed in aorta from both normoglycemic and diabetic animals, these findings suggest that pirfenidone restores endothelial function in aorta from diabetic db/db mice by a mechanism involving K_V_7 channels (Figure 11B).

### Direct Coronary Vasodilator Effect of Pirfenidone

In the present study, micromolar concentrations of pirfenidone induced concentration-dependent relaxations in mouse coronary arteries, while there was no direct relaxant effect in the aorta. In contrast to aorta, inhibitors of NO synthase induce contractions in rat pulmonary arteries and coronary arteries suggesting a large basal release of NO (Symons et al., 2006; Pasgaard et al., 2007). This was also confirmed in porcine and rat coronary arteries by direct measurements using a microsensor for NO (Christensen et al., 2007; Pasgaard et al., 2007). Furthermore, we found that endothelial cell removal and an inhibitor of NO synthase, L-NOARG, abolished pirfenidone relaxations in the mouse coronary arteries suggesting pirfenidone may enhance the effect of NO in the smooth muscle cells of the mouse coronary arteries.

Activation of a series of potassium channels including ATP-sensitive potassium channels, calcium-activated potassium channels, and voltage-gated potassium channels including K_V_7 channels may contribute to vasodilatation in the coronary circulation (Khanamiri et al., 2013; Hedegaard et al., 2014; Lee et al., 2015; Goodwill et al., 2016; Tykocki et al., 2017). To investigate the potassium channel subtypes involved, a combination of blockers of endothelial SK_Ca_ and IK_Ca_ channels, UCL1684 and TRAM-34 were added and reduced pirfenidone relaxation. Since SK_Ca_ and IK_Ca_ channels are involved in the release of NO and initiation of the EDH response, we cannot exclude a direct though minor effect of pirfenidone on this endothelium-dependent vasodilator response. However, blocking the vascular smooth BK_Ca_ channels with TEA or iberiotoxin markedly reduced pirfenidone relaxation suggesting that the major effect of pirfenidone is on potassium channels in the coronary smooth muscle layer. A blocker of K_V_7 channels, XE991 also inhibited relaxations suggesting these channels also contribute to the pirfenidone relaxations in mouse coronary arteries. The NO/cGMP pathway has previously been shown to be coupled either directly or through cGMP to activation of BK_Ca_ channels (Robertson et al., 1993; Bolotina et al., 1994), and has also been suggested to involve voltage-sensitive potassium channels (Sampson et al., 2001; Stott et al., 2015; Mondéjar-Parreño et al., 2019). Therefore, the main effect of pirfenidone is probably that it enhances the effect of NO by modulation of BK_Ca_ and/or Kv7 channels leading to relaxation in mouse coronary arteries. The latter statement is supported by observations in patch clamp studies that revealed pirfenidone in isolated vascular smooth muscle cells increases iberiotoxin-sensitive currents.

### Effect of Pirfenidone on Impaired Endothelium-dependent Relaxation in Diabetic Mice

Several studies have described endothelium-dependent vasodilatation being impaired in patients with type 2 diabetes (Shi and Vanhoutte, 2017). In the aorta of db/db mice, decreased release and response to NO is involved in the impairment of endothelial cell function (Piercy and Taylor, 1998; Miike et al., 2008; Kassan et al., 2014), while in mesenteric small arteries the impaired dilation to acetylcholine was associated with decreased EDH of smooth muscle cells (Chen et al., 2015). Our observations of impaired endothelium-dependent relaxations in the aorta of diabetic db/db mice agree with these findings, and with our recent findings showing impairment of endothelium-dependent relaxation in the aorta from both female and male mice (Beck et al., 2020).

In aorta of diabetic db/db animals, endothelium-dependent relaxations are mainly mediated by NO, while EDH plays a major role in endothelium-dependent relaxations in small resistance arteries (Stankevicius et al., 2011). Pirfenidone improved endothelium-dependent vasorelaxation in the aorta, while there was no effect on acetylcholine relaxation in mesenteric small arteries. NO is the main endothelium-derived factor released by acetylcholine in large arteries, while EDH is the main pathway in acetylcholine relaxation of small mesenteric arteries (Stankevicius et al., 2011). Therefore, our findings in the present study suggest that the effect of pirfenidone is specific, and mainly involves the improvement of endothelium-dependent vasodilatation mediated by NO in large arteries. In contrast to aorta, small mesenteric arteries contribute to vascular resistance, and therefore, our observations that infusion of pirfenidone did not change blood pressure in db/db mice agree with the lack of effect of pirfenidone on small mesenteric arteries. That the effect of pirfenidone is specific, is supported by the observation that pirfenidone did not affect relaxations induced by acetylcholine in the aorta from heterozygous animals, suggesting that the effect is specific for db/db animals with high glucose and impaired endothelium-dependent relaxation. The effect of pirfenidone on acetylcholine relaxation in aorta from diabetic mice was also sex independent, as it was observed in aorta from both male and female db/db mice.

BK_Ca_ channels are involved in acetylcholine-induced NO-mediated relaxation in rabbit aorta (Bolotina et al., 1994) and rat superior mesenteric artery (Climent et al., 2012). In the aorta from both db/db+ and db/db mice, iberiotoxin also inhibited acetylcholine relaxation suggesting BK_Ca_ channels are involved in the endothelium-dependent relaxations. Modulation of BK_Ca_ channels was suggested as a target to treat endothelial cell dysfunction (Félétou, 2009). However, pirfenidone potentiated acetylcholine relaxation independent of the presence of iberiotoxin, a finding suggesting that the effect of pirfenidone on acetylcholine relaxation in the aorta of db/db mice is independent of modulation of BK_Ca_ channels. In contrast to what was observed in the absence of iberiotoxin, pirfenidone potentiated SNP relaxation in aortae in the presence of iberiotoxin. These findings suggest that the large effect of BK_Ca_ channels on SNP relaxation masks the potentiation by pirfenidone in the absence of iberitoxin. Immunoblotting also revealed that the expressions of BK_Ca_ channel subunits are markedly higher compared to expression of K_V_7.4 and K_V_7.5 protein indirectly supporting that activation of BK_Ca_ channels may mask the contribution from K_V_7.4/K_V_7.5 channels in the mouse aorta.

K_V_7 channels regulate vascular tone in many rodent blood vessels, including the mouse aorta (Yeung and Greenwood, 2005; Joshi et al., 2006; Yeung et al., 2007; Mackie et al., 2008; Zhong et al., 2010). There is also evidence that the K_V_7.4 and K_V_7.5 channels are expressed as both homomers and heteromers in vascular smooth muscle (Brueggemann et al., 2011, Brueggemann et al., 2014; Chadha et al., 2014; Jepps et al., 2015). K_V_7 channels were involved in pirfenidone relaxation of mouse coronary arteries. Therefore, we also examined these channels' role in acetylcholine relaxation in the aorta. Acetylcholine relaxation is NO-mediated in mouse aorta (Boedtkjer et al., 2011; Buus et al., 2011). A general blocker of K_V_7 channels, XE991, inhibited relaxations induced by acetylcholine in the aorta from both db/db+ and db/db mice. Previous evidence suggest the cGMP-pathways either indirectly or directly can lead to activation of K_V_7.4/K_V_7.5 channels (Sampson et al., 2001; Stott et al., 2015; Mondéjar-Parreño et al., 2019). Hence our findings suggest the K_V_7.4 and K_V_7.5 channels are involved in the NO-mediated acetylcholine relaxation in the mouse aorta. An observation further supported by immunoblotting showing expression of K_V_7.4 and K_V_7.5 channels in the aorta from both db/db+ and db/db mice.

An opener of K_V_7 channels, flupirtine, causes relaxations in mouse aorta (Yeung et al., 2007). Flupirtine also leftward shifted concentration-response curves for acetylcholine in the aorta from db/db+ and diabetic db/db mice. Flupirtine also potentiated SNP relaxations in the aorta from both db/db+ and db/db mice. These findings suggest that flupirtine by activation of Kv7 channels decreases smooth muscle membrane excitability, thereby potentiating relaxations induced by vasodilators activating the NO/cGMP-pathway.

The effect of the combination of pirfenidone and flupirtine on acetylcholine relaxation in the aorta from db/db mice was not larger than the effect of each drug alone. These findings suggest pirfenidone and flupirtine may potentiate acetylcholine relaxation through the same mechanism. However, pirfenidone did not relax the aorta from db/db mice. There was no potentiating effect of pirfenidone on SNP relaxation in the aorta from male db/db mice. On the other hand, XE991 blocked pirfenidone’s potentiating effect on acetylcholine relaxation in the aorta from diabetic db/db mice. These findings suggest that pirfenidone may involve modulation of signaling mechanisms either down- or upstream of the K_V_7 channels.

Besides voltage, different signal transduction pathways regulate smooth muscle K_V_7 channels, including cyclic AMP, KCNE subunits, and microtubules. Thus, cyclic AMP regulates K_V_7.4 and K_V_7.5 channels in vascular smooth muscle (Khanamiri et al., 2013; Mani et al., 2016; Stott et al., 2016; van der Horst et al., 2020). One may speculate that increased cyclic GMP may increase the cyclic AMP levels in the aorta by inhibiting phosphodiesterase type 3. Still, pirfenidone interactions with phosphodiesterases would be expected to affect both the aorta from db/db+ and diabetic db/db mice.

In the smooth muscle, the Kv7 α-subunits are often regulated by KCNE subfamily ancillary (β) subunits (KCNE1-5). Thus, KCNE4 co-assembles with Kv7.4 and Kv7.5 in vascular smooth muscle cells, and deletion of the KCNE4 subunit impaired β-adrenoceptor relaxation in rat mesenteric arteries (Abbott and Jepps, 2016). Therefore, KCNE4 was also suggested to facilitate Kv7 channel-dependent cAMP relaxations (Jepps et al., 2015; van der Horst et al., 2020). KCNE4 is highly expressed in the mouse aorta compared to other vascular preparations (Yeung et al., 2007) and in the mouse mesenteric arteries, where there is also a high expression of KCNE3 subunits (Abbott and Jepps, 2016). In contrast to the aorta, pirfenidone does not potentiate acetylcholine relaxation in mesenteric arteries suggesting interaction with KCNE4 subunits is an unlikely explanation. However, it would be a relevant mechanism to address. Another acute mechanism regulating K_V_7 channel activity in vascular smooth muscles are microtubules. Thus, disruption of the microtubules with colchicine increased the membrane expression of K_V_7.4 channels and smooth muscle hyperpolarization (Lindman et al., 2018). To the best of our knowledge, there is currently no information regarding interaction of pirfenidone with microtubules. Therefore, further investigation will be required to clarify the mechanisms involved in pirfenidone's potentiating effect on acetylcholine relaxation and how it leads to activation of K_V_7 channels in diabetic db/db mice aorta.

As mentioned in the Introduction, several mechanisms of action have been described to mediate the antifibrotic effect of pirfenidone (Iyer et al., 1999; Hilberg et al., 2012). Scavenging of hydroxyl anions and hydrogen peroxide have also been suggested to be involved in the mechanisms of action of pirfenidone, but millimolar concentrations are required (Misra and Rabideau, 2000; RamachandraRao et al., 2009). In contrast to mannitol, we did not observe a hydroxyl scavenger effect of pirfenidone in therapeutic relevant concentrations. The therapeutic effects of pirfenidone are in the micromolar range (Hilberg et al., 2012). In the present study, we indeed observed effects at endothelium-dependent relaxation at micromolar concentration. This is further supported by our observations that the effect of pirfenidone was specific, as it only potentiated acetylcholine relaxation in the aorta from db/db mice, but not in the aorta from db/db+ mice.

The antifibrotic effect of pirfenidone involve inhibition of the TGFβ signal transduction pathway (Ballester et al., 2020). Increased TGFβ activation is also associated with hypertension and increased myogenic activity in mesenteric arteries (Carnevale et al., 2018). The diabetic db/db mice are not hypertensive (Beck et al., 2020), and pirfenidone did not change endothelium-dependent relaxations in mesenteric arteries from diabetic db/db mice. Therefore, we consider unlikely TGFβ is involved in the positive effect of pirfenidone on vascular tone in the aorta from db/db diabetic mice, although we cannot exclude pirfenidone interfere with signal pathways activated by TGFβ.

### Limitations and Perspectives

Pirfenidone is an antifibrotic drug that increases survival in patients with idiopathic lung fibrosis (Azuma et al., 2005). Moreover, chronic treatment with pirfenidone inhibited angiotensin II and DOCA salt-induced cardiac hypertrophy and fibrosis (Mirkovic et al., 2002; Yamazaki et al., 2012), and in diabetic db/db mice, pirfenidone treatment inhibited mesangial cell proliferation and interstitial collagen formation, though without changing albuminuria (RamachandraRao et al., 2009). The present study shows that pirfenidone restores endothelium-dependent relaxation in the aorta of diabetic db/db mice, but without having an effect in mesenteric small arteries. It remains unclear whether the effect on endothelial function in large arteries will contribute to the antifibrotic effect of pirfenidone. In diabetic patients, there is increased stiffness of the central large arteries (Prenner and Chirinos, 2015). The antifibrotic effect of pirfenidone and the effect on vascular tone may contribute to the conservation of the Wind Kessel function in the large elastic arteries, and hence less backward waves interfering with cardiac function. However, so far these are speculations that can only be addressed by direct measurements with conductance catheters in intact animals (see (Su et al., 2018)). However, our previous studies revealed that aortic remodeling in db/db mice at this age is markedly different from that in diabetic patients (Beck et al., 2020). Therefore, other diabetic animal models or treatment of diabetic patients with pirfenidone will be required to further address whether the effect of pirfenidone on endothelial function in large arteries plays a role in the progression of increased vascular stiffness and organ fibrosis. In addition, further investigation will be required to address whether the direct vasodilator effect in mouse coronary arteries and rat pulmonary arteries is associated with increased flow in these vascular beds.

## Conclusion

In summary, we investigated the effect of the antifibrotic drug, pirfenidone, on vascular tone, and in mouse coronary arteries pirfenidone induced concentration-dependent relaxations, which were inhibited by an inhibitor of NO synthase, high extracellular potassium, and blockers of BK_Ca_ and K_V_7 channels suggesting an involvement of NO and these potassium channels in pirfenidone relaxations. Moreover, pirfenidone increased BK_Ca_ current in vascular smooth muscle. In the aorta from diabetic mice, pirfenidone improves endothelium-dependent vasodilatation a mechanism involving voltage-gated K_V_7 channels. These vascular actions may contribute to the antifibrotic effect of pirfenidone.

## Data Availability

The original contributions presented in the study are included in the article/Supplementary Material, further inquiries can be directed to the corresponding authors.
