# Supplementary material for: Pirfenidone Is a Vasodilator: Involvement of KV7 Channels in the Effect on Endothelium-Dependent Vasodilatation in Type-2 Diabetic Mice
Source: Front Pharmacol. 2021 Jan 12;11:619152. doi: 10.3389/fphar.2020.619152 (PMC7906977; doi:10.3389/fphar.2020.619152)
Supplement: Supplementary file 1 [file datasheet1.pdf]

Supplementary results

INVOLVEMENT OF VOLTAGE-GATED K_V7 CHANNELS IN EFFECT OF PIRFENIDONE ON ENDOTHELIUM-DEPENDENT VASODILATATION IN TYPE-2 DIABETIC (db/db) MICE

Lilliana Beck[1,*], Estéfano Pinilla[1,2], Daniel Arcanjo[1,3], Raquel Hernanz[1, 4], Judit Prat-Duran[1], Asbjørn Petersen[1], Ralf Köhler[5], Majid Sheykhzade[6], Simon Comerma-Steffensen[1,7], and Ulf Simonsen[1, #]

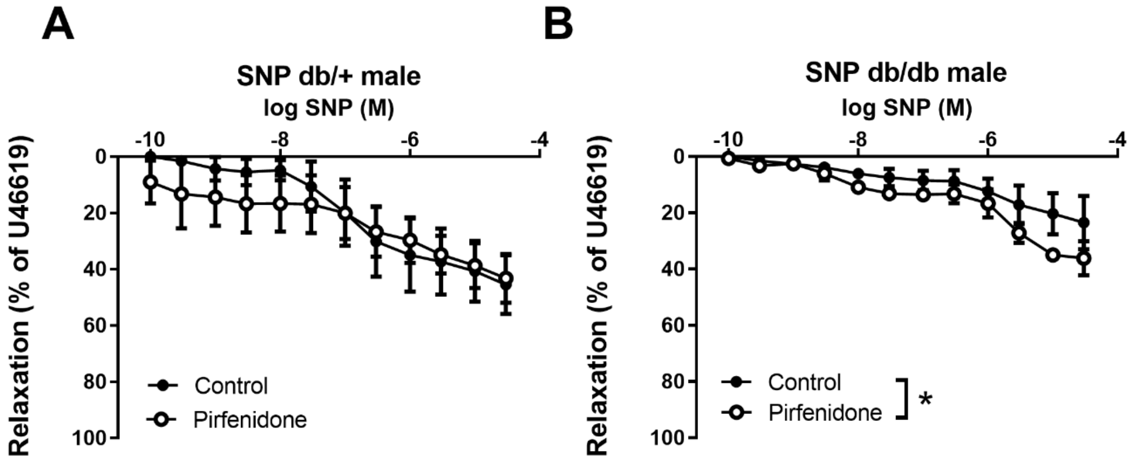

Supplementary Figure S1. Effect of pirfenidone on sodium nitroprusside relaxations in left anterior descending coronary arteries (LAD) from male normoglycemic control (db/db+) and diabetic (db/db) mice. Average relaxations induced by the nitric oxide donor, sodium nitroprusside (SNP) in the absence and the presence of pirfenidone in U46619- contracted coronary arteries from (a) db/db+ (n = 6) (b) db/db mice (n = 3). The results are means ± s.e.mean, where n indicates the number of animals examined. *$P<0.05$ versus control curves, two-way ANOVA.

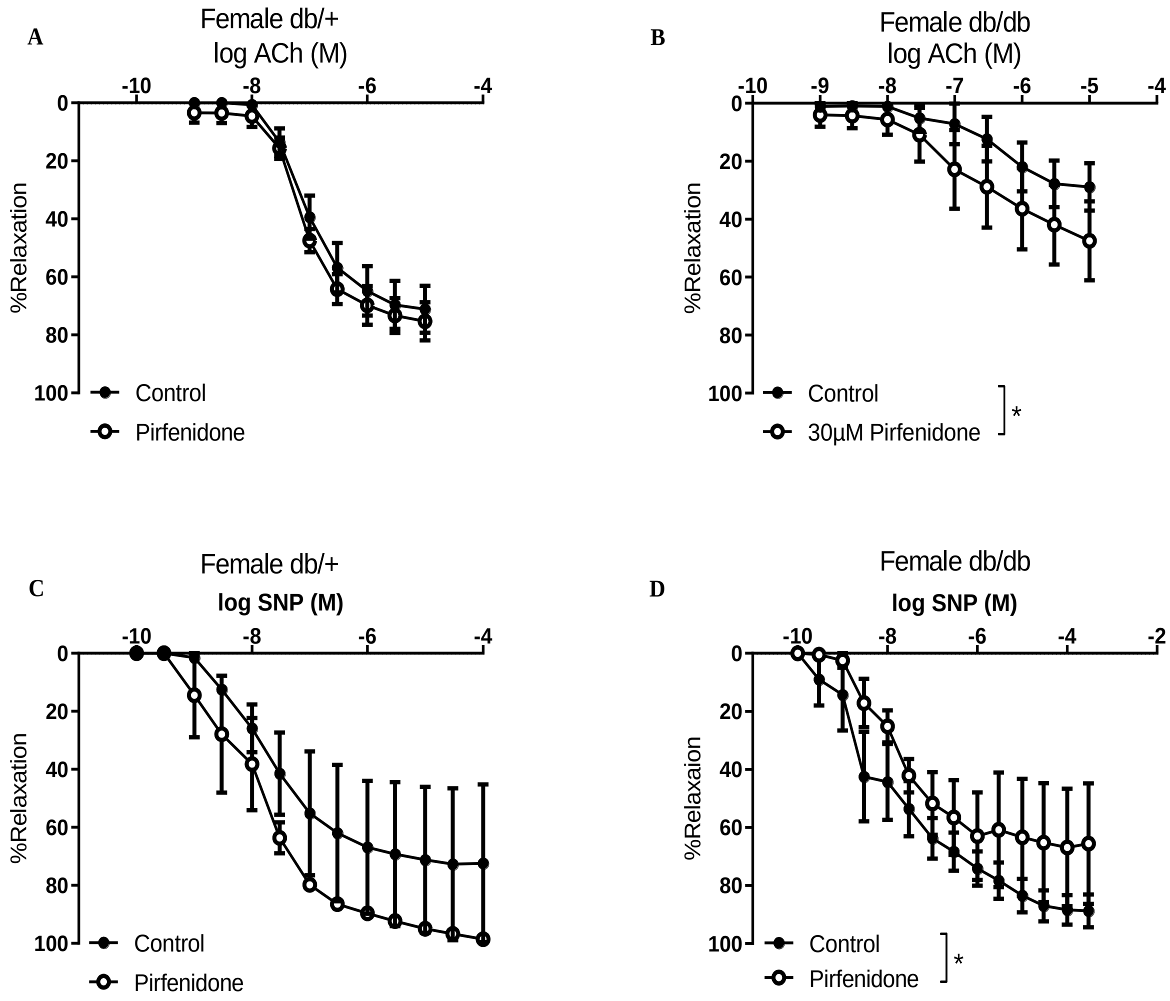

Supplementary Figure S2. Effect of pirfenidone on acetylcholine and sodium nitroprusside relaxations in aorta from female diabetic mice. Pirfenidone enhances acetylcholine relaxation in aorta from female diabetic mice. Average relaxations induced by the endothelium-dependent vasodilator, acetylcholine in the absence and the presence of pirfenidone in phenylephrine-contracted aorta segments arteries from (A) heterozygous control (db/db+) (n = 7) and (B) diabetic (db/db) mice (n = 6). Average relaxations induced by the nitric oxide donor, sodium nitroprusside (SNP) in the absence and the presence of pirfenidone in phenylephrine-contracted aorta segments arteries from (C) db/db+ (n = 6) and (D) db/db mice (n = 5). The results are means ± s.e.mean, where n indicates the number of animals examined. *$P<0.05$ versus respective control curves, two-way ANOVA.

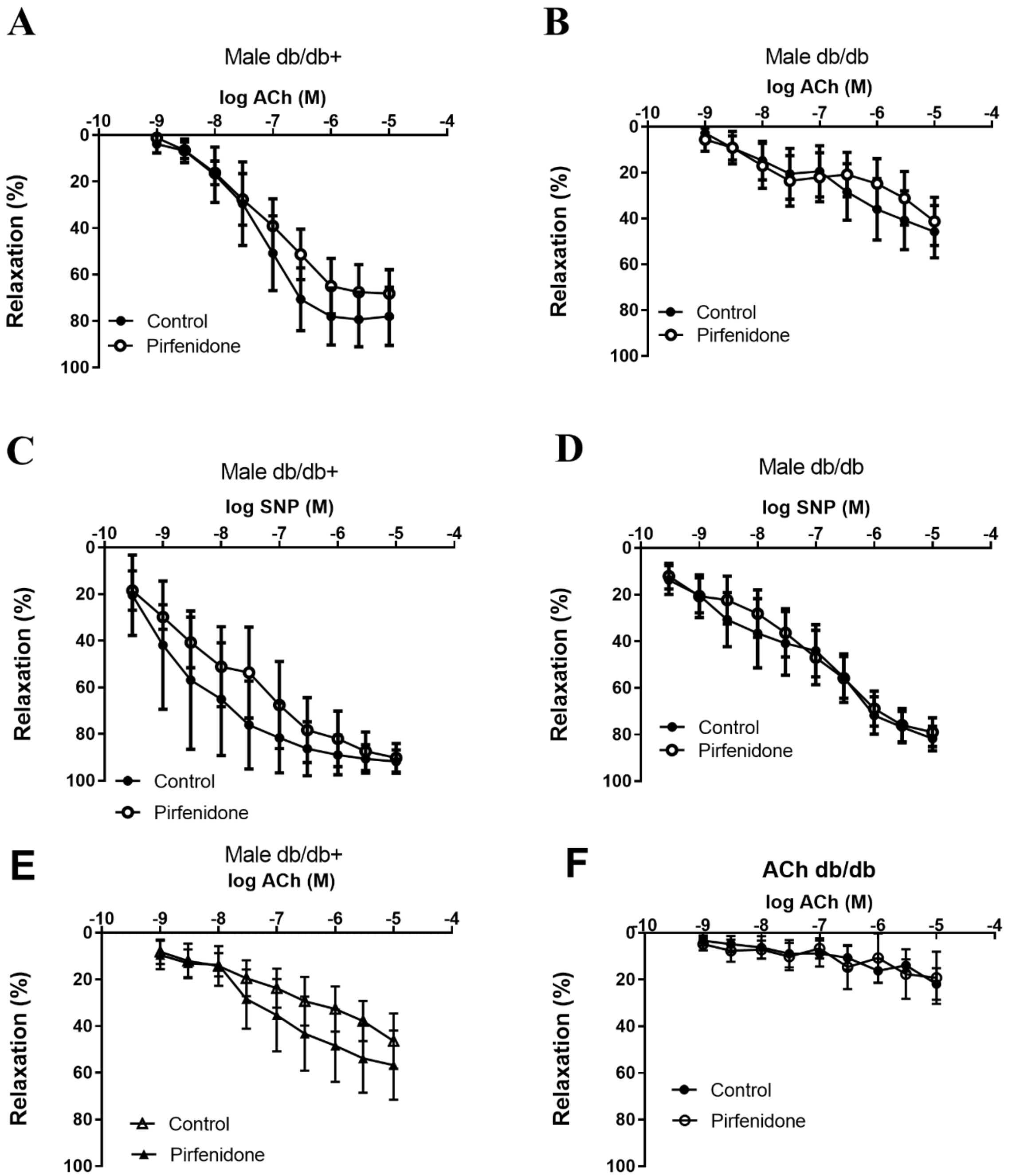

Supplementary Figure S3. Lack of effect of pirfenidone on acetylcholine relaxations in mesenteric small arteries from male diabetic mice. Average relaxations induced by the endothelium-dependent vasodilator, acetylcholine in the absence and the presence of pirfenidone ($3x10^{-5}$ M) in phenylephrine-contracted mesenteric arteries from (A) heterozygous control (db/+) (n =4) and (B) diabetic (db/db) mice (n = 6). Average relaxations induced by the nitric oxide donor, sodium nitroprusside (SNP) in the absence and the presence of pirfenidone in phenylephrine-contracted mesenteric arteries from (C) db/+ (n = 6) and (D) db/db mice (n = 7). Average relaxations to acetylcholine obtained in the presence of L-NOARG plus indomethacin in the absence or the presence of pirfenidone in mesenteric arteries from (E) db/db+ (n=5) and

(F) db/db mice (n=5). The results are means ± s.e.mean, where n indicates the number of animals examined. *P<0.05 versus respective control curves, two-way ANOVA.

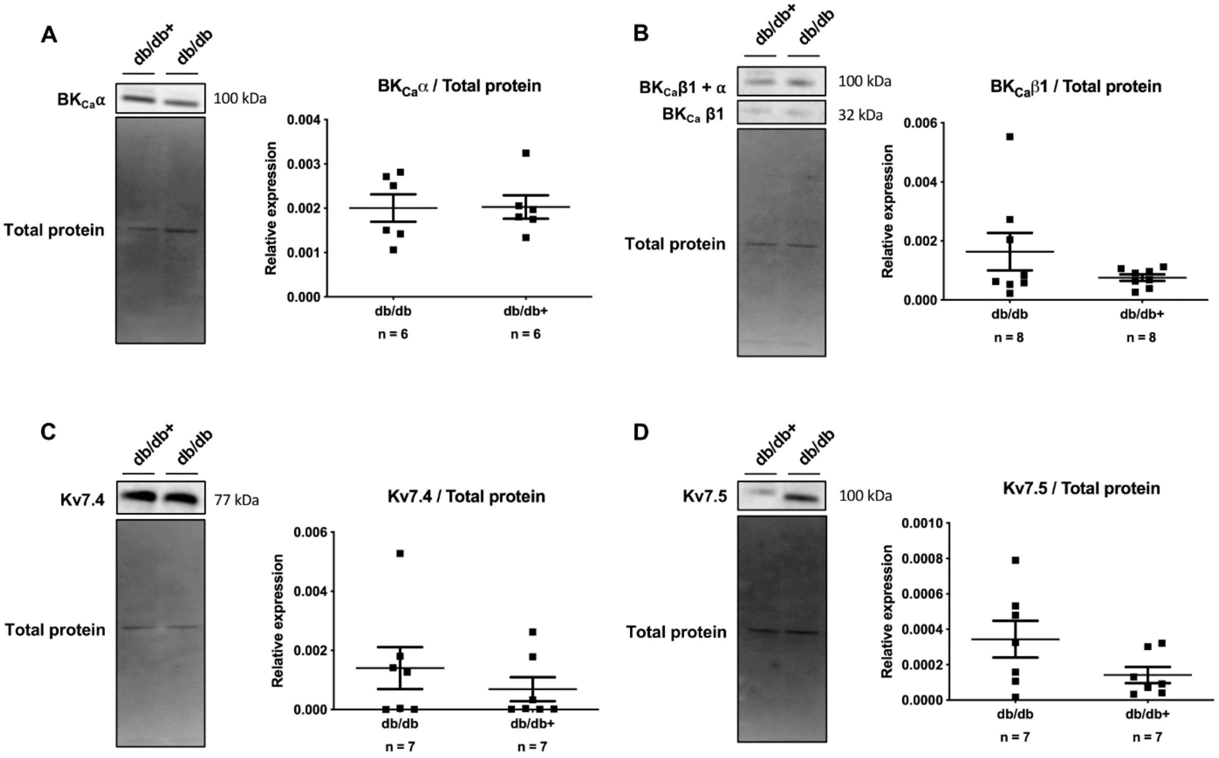

Supplementary Figure S4. Immunoblots in mouse aorta expressed relative to total protein. The Figures show inserts of original immunoblottings and average values expressed as ratio to the total protein for: (A) $BK_{Ca}\alpha$, (B) $BK_{Ca}\beta1$, (C) $K_V7.4$, and (D) $K_V7.5$. The results are means ± s.e.mean from heterozygote db/db+ control mice (n=6) and diabetic db/db (n=6) mice. There were no significant differences.
