# Supplementary material for: Pirfenidone Is a Vasodilator: Involvement of KV7 Channels in the Effect on Endothelium-Dependent Vasodilatation in Type-2 Diabetic Mice
Source: Front Pharmacol. 2021 Jan 12;11:619152. doi: 10.3389/fphar.2020.619152 (PMC7906977; doi:10.3389/fphar.2020.619152)
Supplement: Supplementary file 2 [file datasheet2.pdf]

Western Blot PDF for Review

INVOLVEMENT OF VOLTAGE-GATED K_V7 CHANNELS IN EFFECT OF PIRFENIDONE ON ENDOTHELIUM-DEPENDENT VASODILATATION IN TYPE-2 DIABETIC (db/db) MICE

Lilliana Beck[1,*], Estéfano Pinilla[1], Daniel Arcanjo[1,2], Raquel Hernanz[1, 3], Judit Prat-Duran[1], Asbjørn Petersen[1], Ralf Köhler[4], Majid Sheykhzade[5], Simon Comerma-Steffensen[1,6], and Ulf Simonsen[1, #]

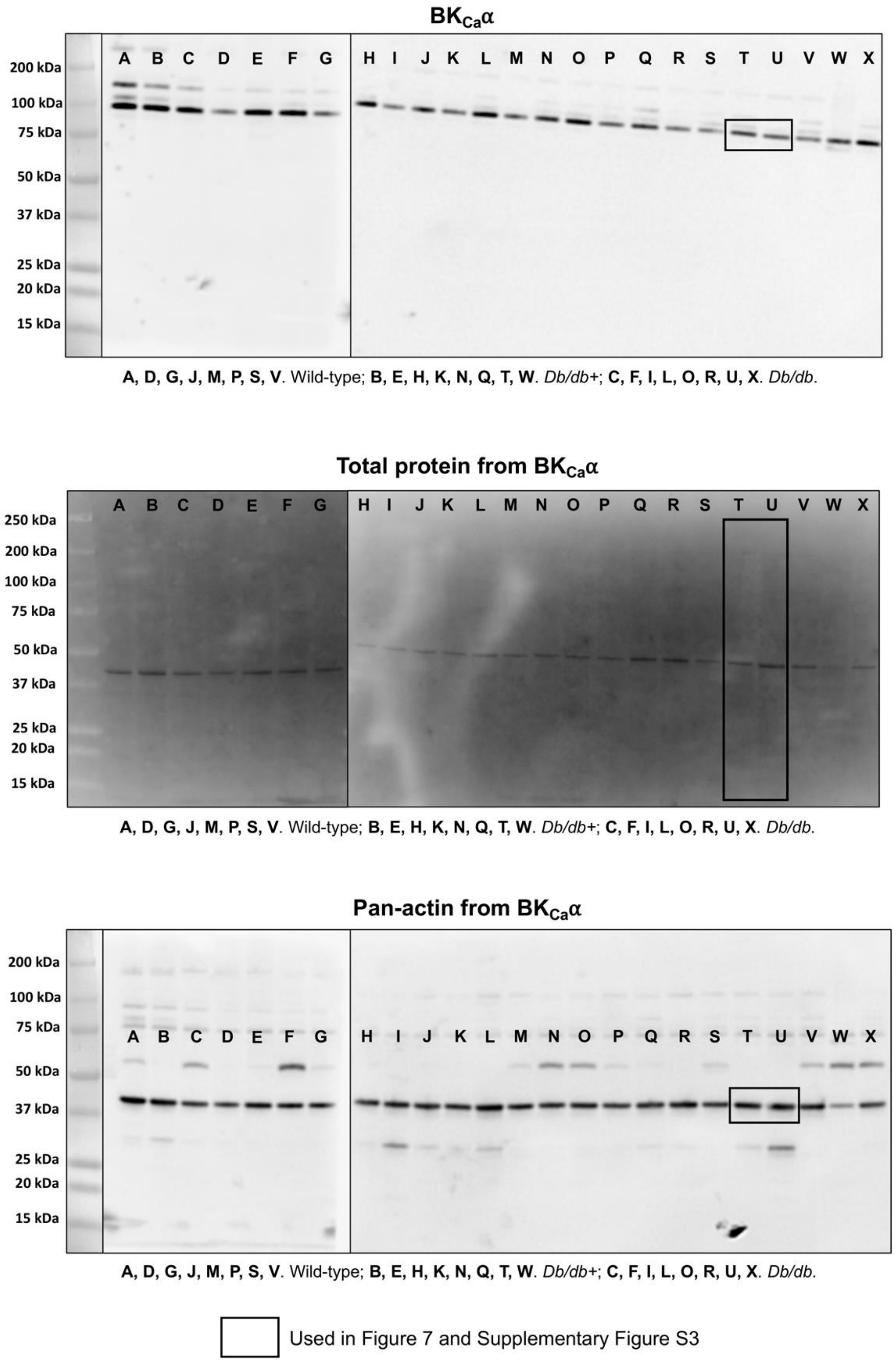

Supplementary Figure S5. Full immunoblot shown for the BKCaα subunit in mouse aorta from db/db+ and db/db mice.

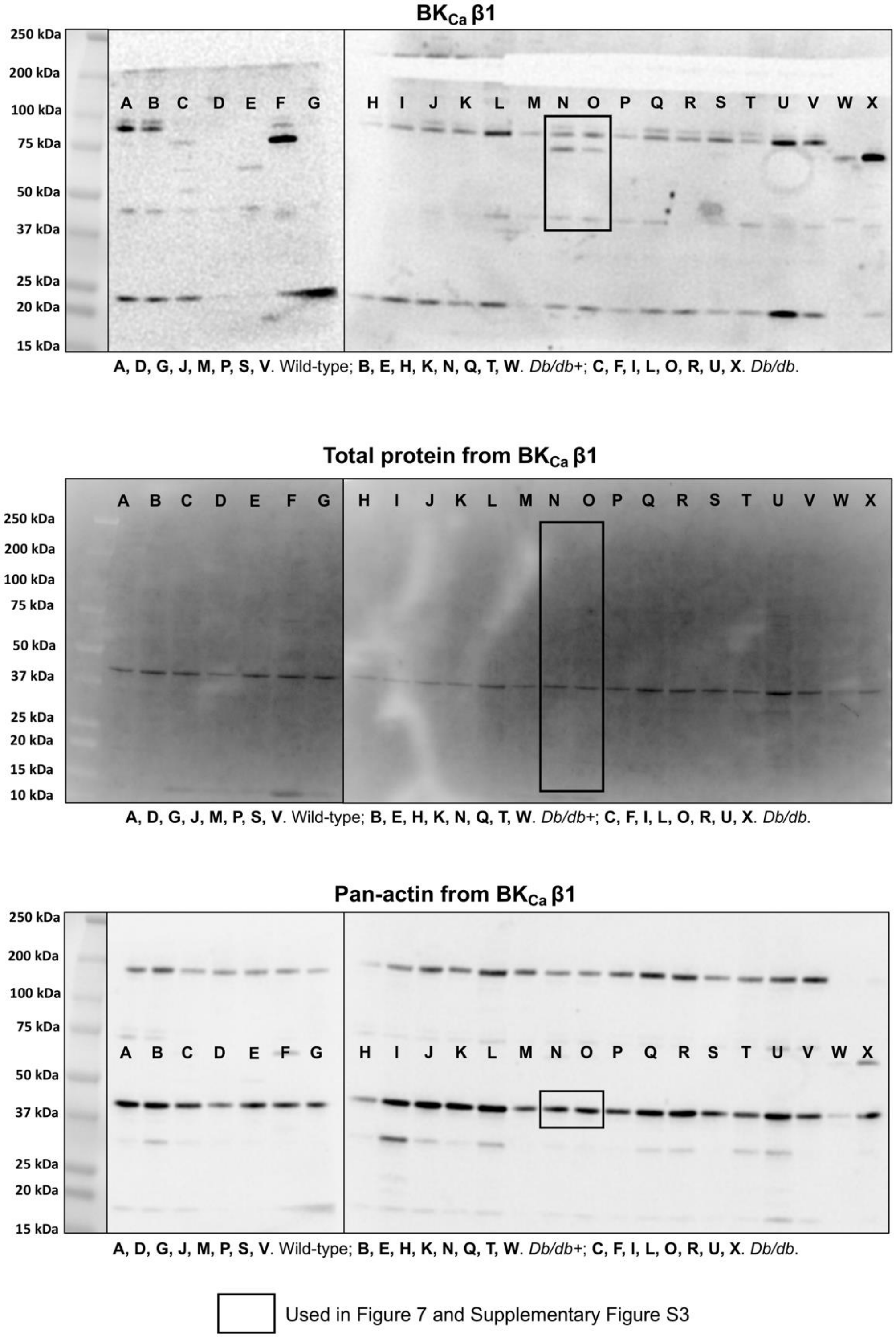

Supplementary Figure S6. Full immunoblot shown for the BKCaβ1 subunit in mouse aorta from db/db+ and db/db mice.

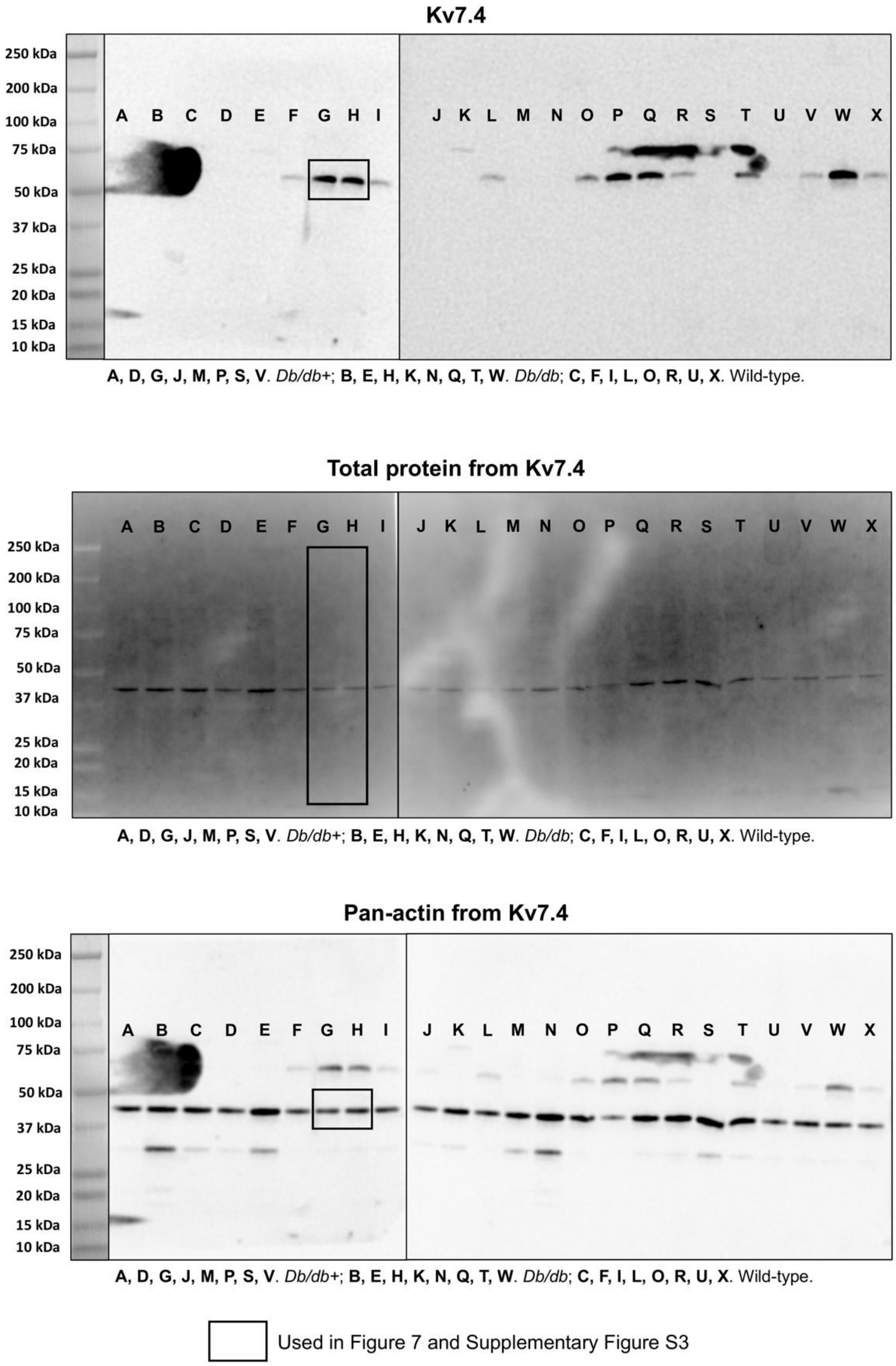

Supplementary Figure S7. Full immunoblot shown for KV7.4 channel in mouse aorta from db/db+ and db/db mice.

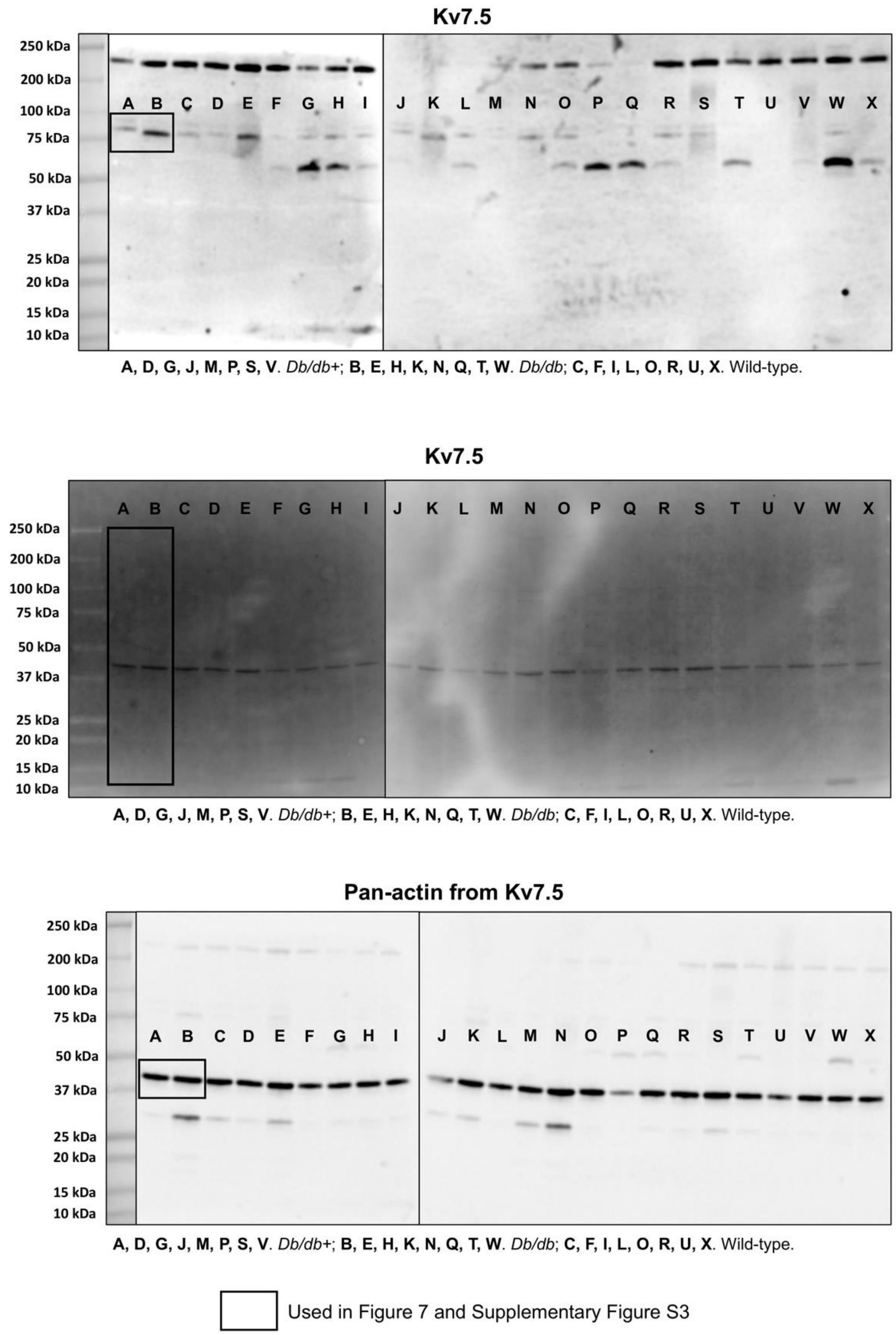

Supplementary Figure S8. Full immunoblot shown for KV7.5 channel in mouse aorta from db/db+ and db/db mice.
